# Combination of transcriptome and Mendelian inheritance reveals novel prognostic biomarker of CTLA-4-related lncRNAs and protective role of nitrogen metabolism pathway in lung adenocarcinoma development

**DOI:** 10.1186/s12885-024-12777-7

**Published:** 2024-08-14

**Authors:** Huisi Shan, Xiaocong Wang, Fei Yin, Yiting Zhou, Liuhan Mao, Xiao Zhu, Caixin Liu

**Affiliations:** 1 Department of Clinical Laboratory, The First Affiliated Hospital of Wannan Medical College (Yijishan Hospital of Wannan Medical College), Wuhu, China; 2 Department of Radiation Oncology, Guangdong Second People’s Hospital, Jinan University, Guangzhou, China; 3 Department of Pathology, Qingdao Municipal Hospital Group, Qingdao, China; 4 Department of Clinical Laboratory, Qingdao Sixth People’s Hospital, Qingdao, China; 5 The Second Affiliated Hospital, Guangdong Medical University, Zhanjiang, China; 6 Department of Internal Medicine, The Seventh Affiliated Hospital of Sun Yat-Sen University, Shenzhen, China; 7 The Marine Biomedical Research Institute of Guangdong Zhanjiang, School of Ocean and Tropical Medicine, Guangdong Medical University, Zhanjiang, China

**Keywords:** CTLA-4-related lncRNA-based signatures, Tumor microenvironment, Lung adenocarcinoma, Prognosis, Mendelian randomization

## Abstract

**Objective:**

Since in the cancer setting, tumor cells may use cytotoxic T-lymphocyte-associated protein 4 (CTLA-4) to evade the immune system. This study aimed to identify CTLA-4-related long non-coding RNAs (lncRNAs) and assess their roles in lung adenocarcinoma (LUAD) development.

**Methods:**

Clinical and genomic data were obtained from The Cancer Genome Atlas (TCGA), MSigDB and Gene Weaver. CTLA-4-related lncRNA-based gene signatures (CTLA4LncSigs) were identified using Cox regression, establishing a risk score model and an independent prognostic model. Enrichment analysis (GO/KEGG) was performed. Mendelian randomization (MR) analysis investigated the nitrogen metabolism and lung cancer relationship, with Bayesian weighted MR (BWMR) addressing uncertainties. Correlations with tumor microenvironment and drug sensitivity were explored.

**Results:**

Nineteen CTLA4LncSigs significantly influenced LUAD prognosis. The risk score demonstrated independence as a prognostic factor. Functional analysis revealed lncRNAs' impact on nitrogen metabolism. MR and BWMR confirmed the protective role of the nitrogen metabolism pathway in lung cancer.

**Conclusion:**

Our study identifies CTLA-4-related lncRNAs associated with LUAD prognosis and uncovers a previously undiscovered protective role of the nitrogen metabolism pathway in combating LUAD development, providing new insights into potential therapeutic targets and prognostic biomarkers for this aggressive cancer subtype.

**Supplementary Information:**

The online version contains supplementary material available at 10.1186/s12885-024-12777-7.

## Introduction

Cytotoxic T-lymphocyte-associated protein 4 (CTLA-4) is an immune checkpoint molecule expressed primarily on the surface of T cells. It regulates the immune response by inhibiting T-cell responses to prevent excessive immune responses [1]. Tumor cells may use CTLA-4 to evade the immune system. Recently, a new type of immunotherapy mediated by immune checkpoint inhibitors (ICIs) has emerged [2], which enhances the lethality of the adaptive immune system against cancer cells. Immune checkpoints are a set of cell surface receptors that suppress T cell functions in an activated state [3]. The Food and Drug Administration (FDA) has approved ICIs designed to target CTLA-4 as a treatment option for various cancer types [4, 5]. In 2021, M Reck et al. demonstrated that Nivolumab + ipilimumab + two cycles of chemotherapy are more effective than conventional chemotherapy for NSCLC patients [6].

The clinical benefit of therapeutic regimens can be predicted by biomarkers in addition to imaging and pathological examination [7]. Biomarkers can be measured in peripheral blood and stool [8, 9], and they have several potential advantages over tissue biopsy, such as being relatively noninvasive and repeatable [10]. Long non-coding RNAs (lncRNAs) play key roles in many cellular processes, including transcription, post-transcriptional regulation, and the cell cycle [11, 12].

Gathering evidence increasingly indicates that lncRNAs play a pivotal role in the development, advancement, and response to treatment in lung cancer [13–15], thereby offering valuable insights for clinical management. It has been predicted that specific lncRNAs regulate CTLA-4 thereby affecting breast cancer progression [16]. However, to date, no studies have investigated the prognostic value of CTLA-4-related lncRNA-based gene signatures (CTLA4LncSigs) in lung adenocarcinoma (LUAD) patients. In this study, we conducted a risk score model and an independent prognostic model. We gain insight into the expression and function of specific CTLA-4 related lncRNAs, evaluating their impact on immune regulation and tumor progression and putting forward some potential drugs. Furthermore, we discussed the protective role of nitrogen metabolism in LUAD by Mendelian randomization (MR) analysis. These validated our findings in transcriptome implying that CTLA4LncSigs is a noval prognostic biomarker of LUAD.

## Experimental approach and procedures

### Acquiring and refining data

For this study, we obtained five gene sets consisting of 405 genes associated with CTLA-4 from the Molecular Signatures Database (MSigDB), accessible at (https://www.gsea-msigdb.org/gsea/msigdb). The immunologic signature collections of genes, namely “GSE37563_WT_VS_CTLA4_KO_CD4_TCELL_D4_POST_IMMUNIZATION_DN” (n = 183) and “GSE37563_WT_VS_CTLA4_KO_CD4_TCELL_D4_POST_IMMUNIZATION_UP” (*n* = 165), were included, along with the curated gene sets “BIOCARTA_CTLA4_PATHWAY” (*n* = 22), “REACTOME_CTLA4_INHIBITORY_SIGNALING” (*n* = 21) and “WP_CANCER_IMMUNOTHERAPY_BY_CTLA4_BLOCKADE” (*n* = 14). After eliminating duplicates, we obtained a total of 382 genes. Additionally, we obtained 90 CTLA-4-related genes from Gene Weaver (https://geneweaver.org), which, when integrated with the 382 genes from MSigDB, yielded a total of 455 genes after removing duplicates [17]. We employed the "limma" package in R software (version 4.0.2; https://cran.r-project.org/) to identify lncRNAs exhibiting a correlation coefficient of 0.4 and *p* < 10^–53^ with the gene sets. Subsequently, we employed univariate Cox regression analysis to screen out significant (*p* < 0.05) lncRNAs (*n* = 131). We acquired gene expression, clinicopathological, and prognostic data for 494 LUAD patients from The Cancer Genome Atlas (TCGA). What’s more, we retrieved and obtained genetic data on blood urea nitrogen levels (ebi-a-GCST90103632, Sample size 173,149, Number of SNPs 7,777,636) and lung cancer (ebi-a-GCST004748, Sample size 85,716, Number of SNPs 7,857,154) via IEU Open GWAS project (https://gwas.mrcieu.ac.uk/) (Table S1).

### Data grouping and prognostic analysis

To pinpoint lncRNAs linked to the overall survival (OS) of LUAD patients, we built a multivariate Cox regression analysis model based on univariate Cox regression analysis and the least absolute shrinkage and selection operator (LASSO) Cox regression analysis, which we called the risk score model. In this model, we can not only get CTLA4LncSigs, but also calculate the risk score of each patient. We also used this model to group 494 patients from the TCGA to ensure that there were no statistical differences in each clinicopathological factor between the training and testing datasets.

The risk score for each patient was computed utilizing the following formula in the model: $Risk\,score=\sum_{i=1}^{n}coeflncRNAi\times \text{exp}\left(coef\right)lncRNAi$ (coef indicates the regression coefficients, exp (coef)) indicates the expression level in LUAD patients) [18, 19]. By employing the median risk score as the threshold, we segregated LUAD patients in the training dataset into two distinct subgroups: the high-risk group and the low-risk group. Subsequently, we evaluated the OS of these two subgroups using the Kaplan–Meier method.

This study delved into exploring the correlation between the risk score and various clinicopathological factors, including gender, age, American Joint Committee on Cancer (AJCC) stages, primary tumor (T), regional lymph nodes (N), and distant metastasis (M). To assess the impact of these factors and risk score on patient prognosis, we utilized univariate and multivariate Cox regression analyses to develop an independent prognostic model. The performance of this model was evaluated using receiver operating characteristic (ROC) curves and concordance index (C-index) curves. Furthermore, we employed a nomogram to visualize how to predict a patient's survival probability, which was assessed by the calibration curve. Moreover, we used the Kaplan–Meier method to compare the survival time of patients in the high-risk and low-risk groups with different clinicopathological factors [20, 21], so as to verify whether the risk score is predictive. Additionally, we performed principal component analysis (PCA) to investigate genetic differentiation between high-risk and low-risk subgroups.

### The analyses encompassed the enrichment of gene ontology and the Kyoto encyclopedia of genes and genomes

To gain a deeper understanding of the role played by CTLA4LncSigs, we conducted a screening process to identify lncRNAs exhibiting significant differences in expression levels (|logFC|> 1, *p* < 0.05) between high-risk and low-risk subgroups. Subsequently, we carried out enrichment analyses for Gene Ontology (GO) and the Kyoto Encyclopedia of Genes and Genomes (KEGG) pathways using R packages. In the Fisher exact test, the p-values for GO and KEGG were corrected for false discovery rate (FDR) and found to be below 0.05 and 0.3, respectively, signifying the significance of these indicators.

### Analysis of tumor microenvironment

We employed Gene Set Variation Analysis (GSVA) to examine CTLA4LncSigs and assess the disparity in immune function-related expression associated with CTLA4LncSigs between the high-risk and low-risk subgroups.

Tumor Mutation Burden (TMB) quantifies the cumulative count of somatic gene coding errors, base substitutions, gene insertions, or deletions identified per million base pairs [22]. Several investigations have provided evidence that individuals with elevated TMB experience notably increased response rates and extended periods of progression-free survival compared to individuals with lower TMB levels [23]. We conducted a comparison of TMB between the high-risk and low-risk subgroups and illustrated the outcomes through survival curves.

Next, we analyzed tumor immune escape and immunotherapy of CTLA4LncSigs using the Tumor Immune Dysfunction and Exclusion (TIDE) web platform, which can infer the role of genes in regulating tumor immunity and evaluate biomarkers to predict ICI clinical response. In particular, we acquired TIDE scores for 494 patients diagnosed with LUAD using the TIDE web platform (http://tide.dfci.harvard.edu/). Our analysis encompassed a range of immune biomarkers and cell factors, which included IFNG [24], Microsatellite Instability (MSI) score [25], Merck18, CD274/PD-L1 [26], CD8 [27], Myeloid-derived suppressor cell (MDSC) [28], immune exclusion, immune dysfunction, cancer-associated fibroblasts (CAF) [29] and tumor-associated macrophages M2 (TAM M2) [30].

### Exploring available drugs

To identify potential drug candidates for LUAD patients, we employed the "pRRophetic" package within R software to estimate the semi-inhibitory concentration (IC50) of a variety of chemotherapy drugs sourced from the Genomics of Drug Sensitivity in Cancer (GDSC) database. We then analyzed the drug responses in both the high-risk and low-risk subgroups.

### Mendelian randomization analysis

Nitrogen metabolic pathways derived from KEGG are closely related to CTLA-4LncSigs. To assess the link between CTLA-4LncSigs and LUAD, we explored whether there is a relationship between nitrogen metabolic pathways and LUAD. The data from IEU Open GWAS project were subjected to a 2-sample Mendelian randomization (2SMR) analysis [31]. We used the inverse-variance weighted (IVW) method as the leading analysis while the MR-Egger regression method was used to perform the pleiotropic test. Leave-One-Out Mendelian Randomization (LOO MR) was used to assess the impact of each SNP on the outcome. We took into account the uncertainty of the estimated weak effects and the possible bias in MR analysis through Bayesian weighted Mendelian randomization (BWMR) based on the following formula: $\text{P}\left(\beta |data\right)=\frac{P\left(data|\beta \right)P\left(\beta \right)}{P\left(data\right)}$ ($\beta$ indicates the causal effects of genes on exposure and outcomes.) [32].

### Statistical analysis

Statistical analysis and figure plotting were conducted using the R software. The OS was presented through the Kaplan–Meier curve. A significance level of* p *< 0.05 was used to denote statistical significance.

## Results

### Identifying lncRNAs associated with CTLA-4

Using univariate Cox regression analysis, we identified 131 lncRNAs with p values less than 0.05, among which LINC01537, SMILR, AC025419.1, and AC108136.1 were significantly associated with LUAD (*p* < 10^–6^) (Table S2).

### Risk and prognosis analysis of CTLA4LncSigs

**Fig. 1:**
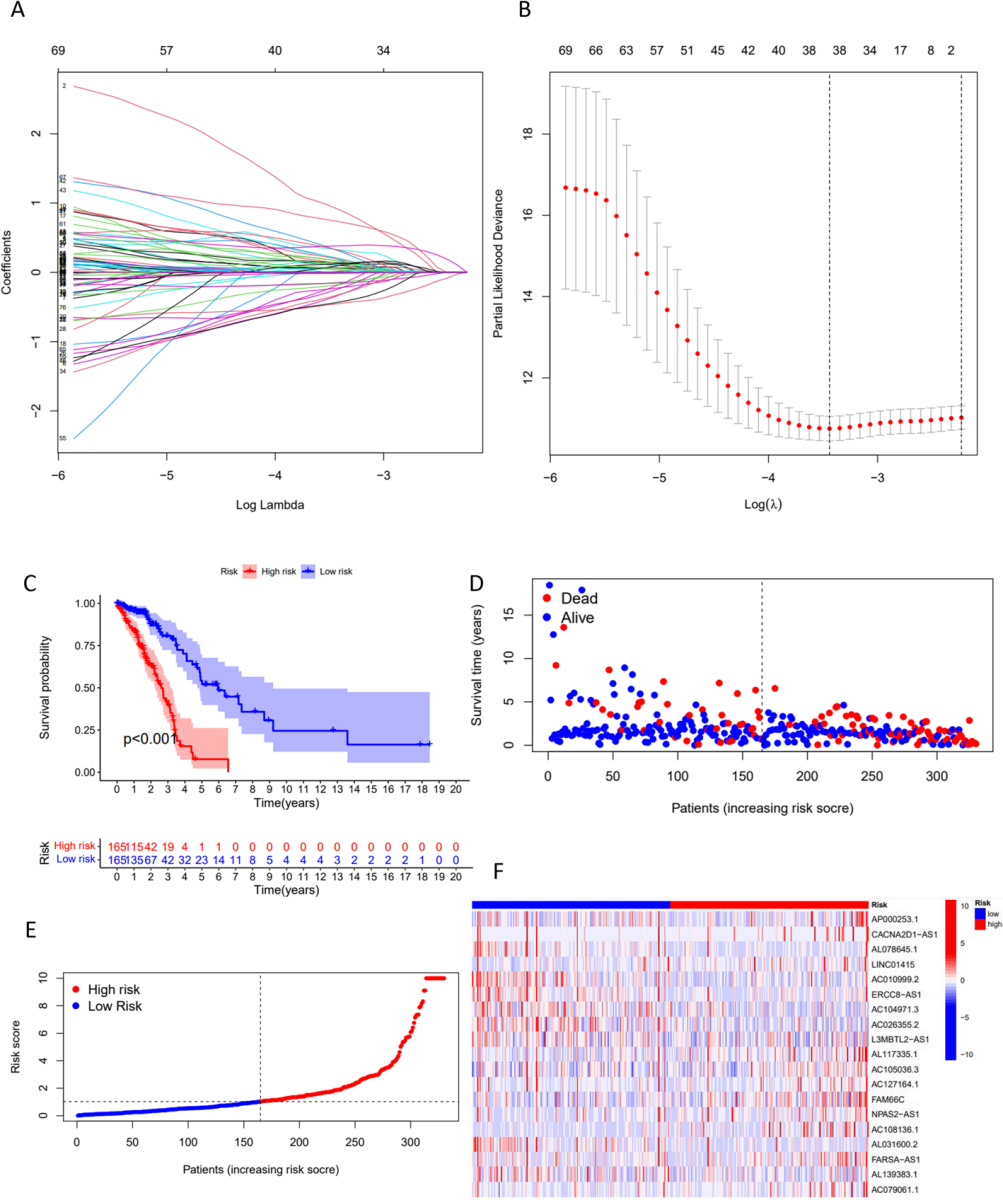
Results of the LASSO Cox regression analysis and Kaplan–Meier method in the training dataset. In the training dataset, the best penalty parameter (λ) was obtained by minimum criteria via tenfold cross-validation (**A**). The LASSO coefficient profile of 38 CTLA4LncSigs (**B**). Longer survival times and higher survival rates of patients in the high-risk subgroup were calculated by the Kaplan–Meier method in the training dataset. The overall survival curves (**C**). The distribution of risk scores (**D**). The survival status (**E**). The heat map showed CTLA4LncSig’s expression in the training dataset (**F**)

We randomly divided the 494 LUAD patients into two groups: a training set (*n* = 330) and a testing set (*n* = 164). We confirmed that there were no statistically significant differences (p > 0.05) in clinical characteristics between these two datasets (Table S3). Through the univariate Cox regression analysis (Table S4), LASSO Cox regression analysis (Fig. 1A-B) and multivariate Cox regression analysis (Table S5) in the risk score model, we identified 19 prognostic CTLA4LncSigs. This set comprised 8 lncRNAs with a positive correlation (hazard ratio, HR < 1) and 11 lncRNAs with a negative correlation (HR ≥ 1) (Table S5). Using the formula previously established ($Risk\,score=\sum_{i=1}^{n}coeflncRNAi\times \text{exp}\left(coef\right)lncRNAi$ (coef indicates the regression coefficients, exp (coef)) indicates the expression level in LUAD patients)), we calculated the risk score for each patient within the training dataset. Subsequently, we categorized them into two subgroups: high-risk (*n* = 165) and low-risk (*n* = 165), based on the median risk score. The Kaplan–Meier analysis demonstrated that patients belonging to the high-risk subgroup exhibited significantly lower survival time and rate in comparison to those in the low-risk subgroup (*p* < 0.001) (Fig. 1C-F). The heatmap of the training dataset demonstrated differential expression of CTLA4LncSigs between high-risk and low-risk subgroups. AC010999.2, ERCC8 − AS1, and AC104971.3 were highly expressed in the low-risk subgroup, indicating their tumor-protective role. Conversely, AP000253.1 and FAM66C were highly expressed in the high-risk subgroup, indicating their role as tumor risk factors (Fig. 1F).

We assessed the precision of the outcomes obtained from the training dataset by examining the testing (*n* = 164) and entire datasets (*n* = 494) using the same approach. At the outset, we stratified both the testing dataset (*n* = 84) and the entire dataset (*n* = 249) into high-risk subgroups, along with low-risk subgroups (testing dataset *n* = 80; entire dataset i = 245), using the median risk score as the criterion. The Kaplan–Meier survival curves for both the testing dataset (*p* = 0.019) and the entire dataset (*p* < 0.001) mirrored those observed in the training dataset, affirming that individuals in the high-risk subgroup experienced notably shorter survival times compared to those in the low-risk subgroup (Figure S1A-B). Furthermore, the survival rate among LUAD patients within the high-risk subgroup was notably lower than that within the low-risk subgroup, a trend consistently observed in both the testing and entire datasets. These findings align with the results obtained from the training dataset (Figure S1C-F). The heatmap of the testing and entire datasets revealed that AC010999.2, ERCC8 − AS1, and AC104971.3, the protective factors, were highly expressed in low-risk areas, while AP000253.1 and FAM66C, the risk factors, were highly expressed in high-risk areas (Figure S1G-H). These findings suggest that the risk score can accurately predict the survival outcome of LUAD patients.

### Relationship between risk score and clinicopathological factors

According to the risk score model we had developed previously, we calculated the risk score of each patient through following formula:  $Risk\,score=\sum_{i=1}^{n}coeflncRNAi\times \text{exp}\left(coef\right)lncRNAi$ (coef indicates the regression coefficients, exp (coef)) indicates the expression level in LUAD patients). Patients were stratified into high-risk and low-risk groups based on the median risk score. We further investigated the relationship between clinicopathological factors and risk score and found that the later the clinical stage of the tumor, the higher the risk score (Figure S2A-C). There was a positive correlation between the risk score and the survival status of patients (Figure S2D). However, age (*p* = 0.8), race (*p* > 0.05), gender (*p* = 0.5), and M (*p* = 0.7) had no significant associations with risk score (Figure S2E-H). We infer that the sample size and statistical power of our study might not be sufficient to detect subtle differences linked to these clinicopathological factors. Larger cohorts could help elucidate these associations more clearly.

### Establishment and evaluation of independent prognostic model

**Fig. 2:**
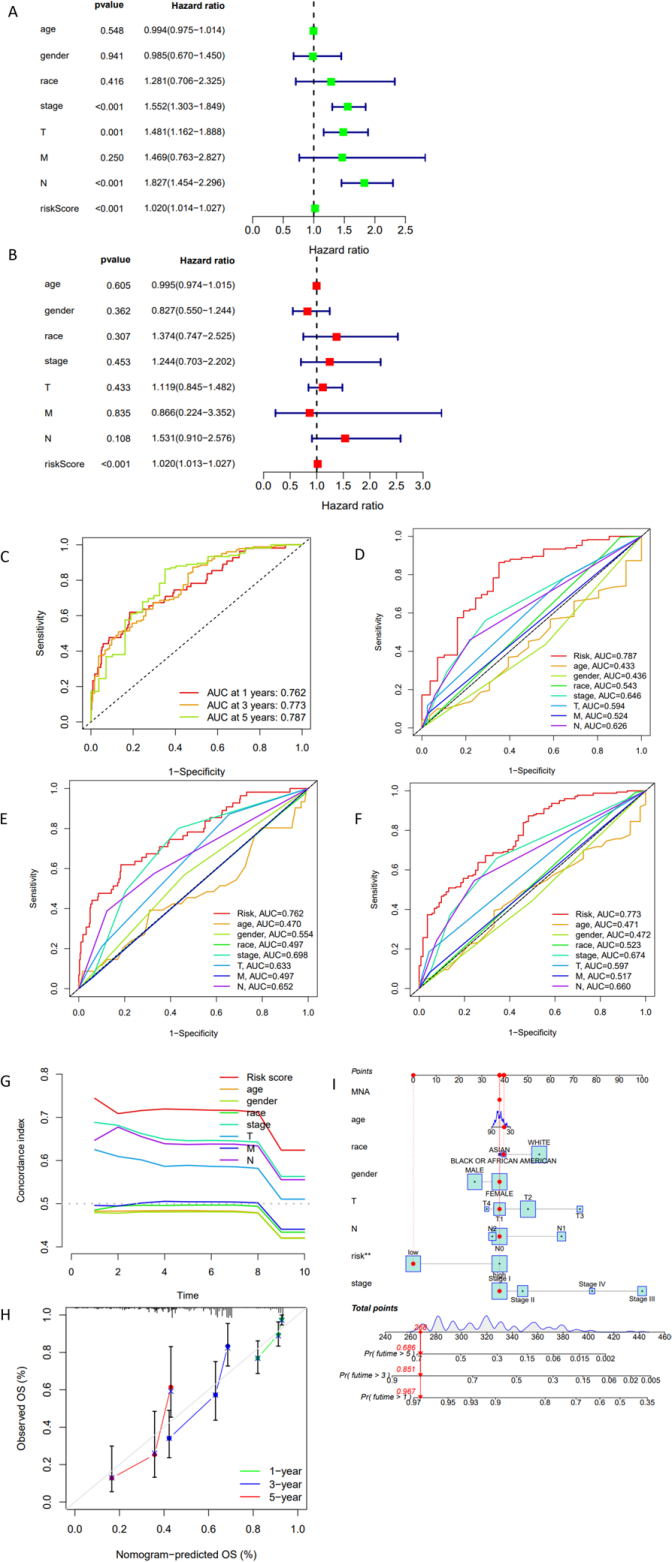
The Result of Independent Prognostic Model in Entire Dataset. **A** is the forest map of univariate Cox regression analysis. **B** is the forest map of multivariate Cox regression analysis. The ROC curve of independent prognostic model for predicting 1, 3 and 5 years survival rates (**C**). The ROC curves of each clinicopathological factor and risk score for predicting 1 (**E**), 3 (**F**), 5 (**D**) years survival rates. The C-index curve of prognostic factors (**G**). Calibration curve evaluating the nomogram (**H**). The nomogram of predicting patients’ survival rates. “**” means that the P value is less than 0.01 (**I**). The specific steps of using a nomogram are as follows: First, find the position of clinicopathological factors of the patient in the nomogram. Then, find their vertical counterparts on “Points” at the top of the nomogram. The number is the score for an individual factor. Add the scores of all factors to get the “Total points”. “Total points” at the bottom correspond to the 1-year, 3-year, and 5-year survival rates of the patient

Following the application of the independent prognostic model, which was built through both univariate (Fig. 2A) and multivariate Cox regression analysis within the entire dataset, our findings indicated that among variables such as age, gender, race, AJCC stage, T, M, N, and risk score, only the risk score exhibited a statistically significant association (*p* < 0.05) with the survival time of LUAD patients (Fig. 2B).

ROC curve illustrated that the model demonstrated strong predictive performance for estimating the survival rate of LUAD patients (1-year area under the curve, AUC = 0.762, 3-year AUC = 0.773, 5-year AUC = 0.787, as shown in Fig. 2C). When it comes to forecasting the 5-year survival rate of LUAD patients, the following factors exhibited significance with AUC values greater than 0.5: risk score (AUC = 0.787), race (AUC = 0.543), AJCC stage (AUC = 0.646), T status (AUC = 0.594), the M (AUC = 0.524), and N involvement (AUC = 0.626). This is depicted in Fig. 2D. The prognostic determinants for the 1-year and 3-year survival rates are depicted in Fig. 2E, F, respectively. In summary, the risk score emerged as the most robust predictor of LUAD patient survival, with other factors potentially serving as supplementary indicators. The C-index curve indicated that the risk score, AJCC stage, T, and N could still be utilized as predictors of the survival rate of LUAD patients (index > 0.5), whereas the indices of other factors were close to 0.5 (Fig. 2G). This outcome was by the ROC curve's forecast.

We created a nomogram utilizing the independent prognostic model to efficiently estimate a patient’s OS at 1, 3, and 5 years, as depicted in Fig. 2I. The nomogram's reliability was validated through the calibration curve (Fig. 2H).

In general, our independent prognostic model demonstrates strong capability in predicting the prognosis of LUAD patients.

### Validating the reliability of risk score

By comparing the survival times of patients in the high-risk and low-risk subgroups with different clinicopathological factors, we can validate the reliability of risk score. As demonstrated by a series of Kaplan–Meier survival curves (Figure S3), patients in the high-risk subgroup with the following clinicopathological factors had significantly (*p* < 0.05) shorter survival rates than those in the low-risk subgroup: patients under 65 years (*p* = 0.027, Figure S3A), patients over 65 years (*p* < 0.001, Figure S3B), male patients (*p* = 0.003, Figure S3C), female patients (*p* < 0.001, Figure S3D), white patients (*p* < 0.001, Figure S3E), Asian patients (*p* = 0.046, Figure S3T), patients with stage I (*p* = 0.006, Figure S3F) and II (*p* = 0.01, Figure S3G), patients with N0 (*p* < 0.001, Figure S3H) and N1 (*p* = 0.036, Figure S3I), patients with T2 (*p* < 0.001, Figure S3J) and patients with M0 (*p* < 0.001, Figure S3K). However, the survival rate of patients with T1 (*p* = 0.579, Figure S3L), T3 (*p* = 0.089, Figure S3M), and T4 (*p* = 0.644, Figure S3N), patients with stage III (*p* = 0.306, Figure S3O) and IV (*p* = 0.444, Figure S3P), patients with M1 (*p* = 0.444, Figure S3Q), patients with N2 (*p* = 0.696, Figure S3R) and black or African American patients (*p* = 0.071, Figure S3S) were not associated with the risk score. We hypothesize that this is due to the small sample size, e.g., only 13 cases for T4 and 18 cases for both stage IV and M1. Since Asian had only 6 cases, its results also need to be considered carefully. Overall, survival times for high- and low-risk patients differed significantly across most clinicopathological groups. Thus the risk score has prognostic value.

We analyzed differences in LUAD’s genes (Figure S4A), LUAD’s mRNAs (Figure S4B), LUAD’s lncRNAs (Figure S4C), and CTLA4LncSigs (Figure S4D) in high- and low-risk patients by PCA. All these plots showed clear separation between high-risk and low-risk groups. This indicated that the expression profiles of CTLA-4-related lncRNAs can effectively distinguish between these groups, suggesting distinct genetic characteristics associated with different risk levels. The distinct clustering observed in PCA implies underlying genetic heterogeneity between high-risk and low-risk groups. This heterogeneity may be driven by differential regulation of CTLA-4-related lncRNAs, which in turn could influence tumor behavior and patient prognosis. This reinforces the potential of CTLA-4-related lncRNAs as biomarkers for predicting patient outcomes and tailoring personalized treatment strategies.

### Functional annotation and classification of lncRNA GO/KEGG

**Fig. 3:**
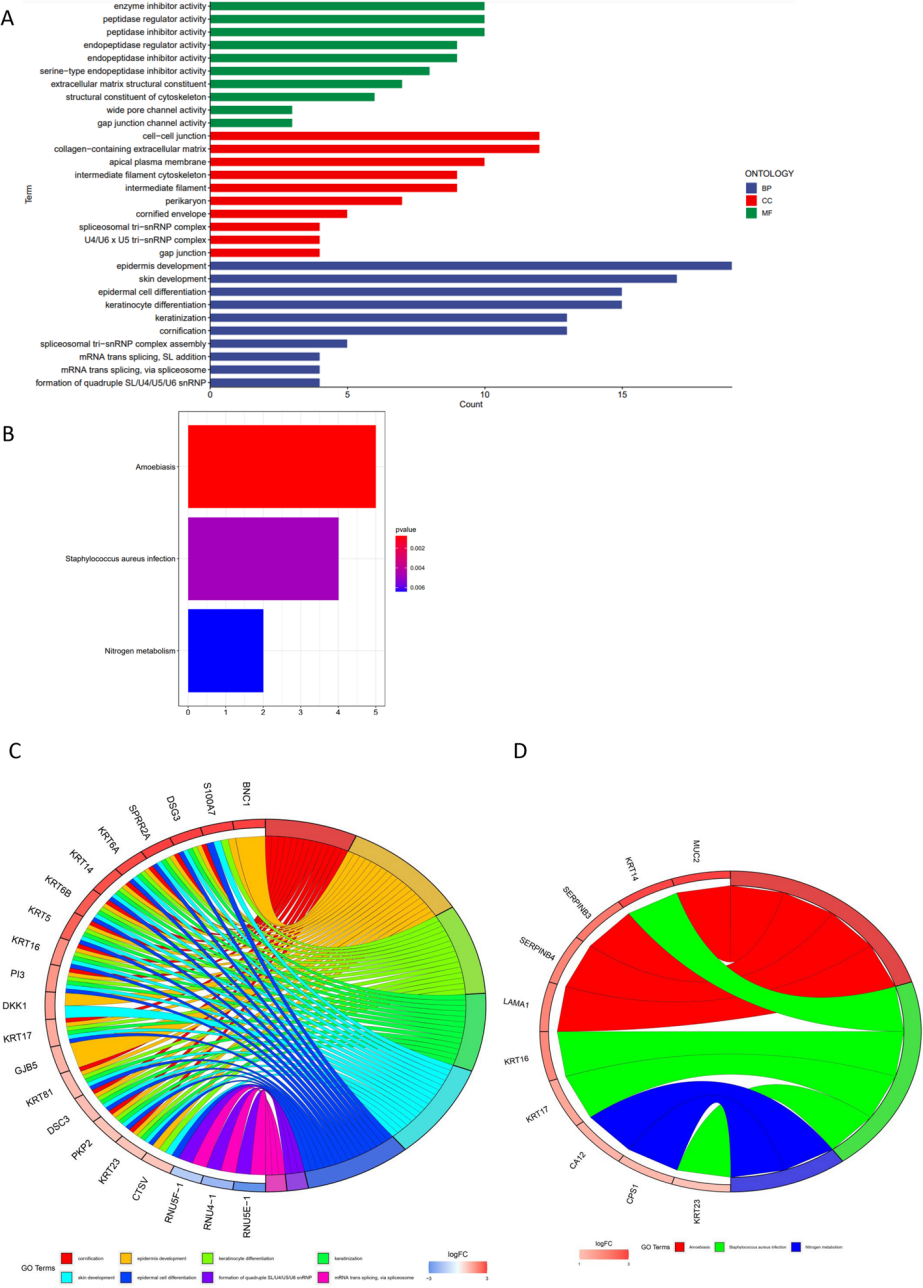
The result of all differential lncRNAs (*n* = 240) between high-risk and low-risk subgroups in GO/KEGG. **A** and **C** are the results of the GO analysis. **B** and **D** are the results of the KEGG analysis

The GO functional analysis encompassed three distinct categories: biological process (BP), cellular component (CC), and molecular function (MF) (Fig. 3A). The findings indicated that among the lncRNAs, 22 BP terms, 25 CC terms, and 12 MF terms exhibited statistical significance (*p* < 0.05, with FDR correction also *p* < 0.05). The mainly enriched BP terms were cornification, epidermis development and aging. The mainly enriched CC terms were cell–cell junction, intermediate filament and apical plasma membrane. The mainly enriched MF terms included alpha-catenin binding, enzyme inhibitor activity and extracellular matrix structural constituent. The relationship between lung cancer and apical plasma membrane and aging has also been found in other study [33]. Extracellular matrix structural constituent was linked to lung metastasis in colorectal cancer [34, 35]. Cell–cell junction is conducive to the collective invasion of tumor cells [36].

Hence, it is plausible that lncRNAs associated with CTLA-4 could potentially contribute to the invasion of lung cancer cells.

Subsequently, KEGG enrichment pathway analysis uncovered 3 significantly (*p* < 0.05, FDR corrected *p* < 0.3) enriched pathways (Fig. 3C), namely Amoebiasis, Staphylococcus aureus infection, and Nitrogen metabolism, with their corresponding lncRNAs shown in Fig. 3D. As cancer tissue has a much higher nitrogen requirement than non-proliferating normal tissue, regulating nitrogen metabolism may represent a potential cancer treatment [37, 38]. In conclusion, the inhibition of (CTLA-4)-related lncRNAs can effectively disrupt the nutrient supply to tumor tissue, thereby inhibiting tumor growth.

### Assessment of immune function in high-risk and low-risk subgroups

The expression levels of lncRNAs associated with 13 immune functions were analyzed using a heat map (Figure S5) in both high-risk and low-risk subgroups. These functions encompassed APC co-inhibition, APC co-stimulation, CCR, checkpoint, cytolytic activity, HLA, inflammation promotion, MHC class I, parainflammation, T cell co-inhibition, T cell co-stimulation, type I IFN response, and type II IFN response [39]. The heatmap showed that cytolytic activity and human leukocyte antigen (HLA) in all datasets were highly expressed in the low-risk subgroup, suggesting that they are the protective factors. Similar findings were shown in patients with squamous cell carcinoma of the oral tongue, where high immune cytolytic activity was associated with a better prognosis [40]. Reduced antigen presentation due to low expression of HLA promotes immune escape [41]. Parainflammation displayed a higher expression in high-risk subgroup which is a procarcinogenic inflammatory process [42]. These observations suggest that immune functions are strong related to risk score.

### Survival analysis of TMB

Additionally, we conducted a comparison of TMB between the high-risk and low-risk subgroups and observed no statistically significant distinctions (*p* < 0.05) in the training dataset (*p* = 0.44), testing dataset (*p* = 0.91), and the entire dataset (*p* = 0.47) (Figure S6A-C). Furthermore, there was no significant relationship (*p* < 0.05) between TMB and OS of LUAD patients in the training dataset (*p* = 0.066), testing dataset (*p* = 0.184), and the entire datasets (*p* = 0.056) (Figure S6D-F). Nonetheless, the combination of TMB and the risk score yielded a notable influence on the OS of LUAD patients (Figure S6G). While the outcomes in the testing dataset (Figure S6H) and entire datasets (Figure S6I) displayed slight variations compared to the training dataset, it remained consistent that patients with high TMB and low-risk scores continued to exhibit the longest survival.

### Analysis of tumor immune escape and immunotherapy of CTLA4LncSigs

**Fig. 4:**
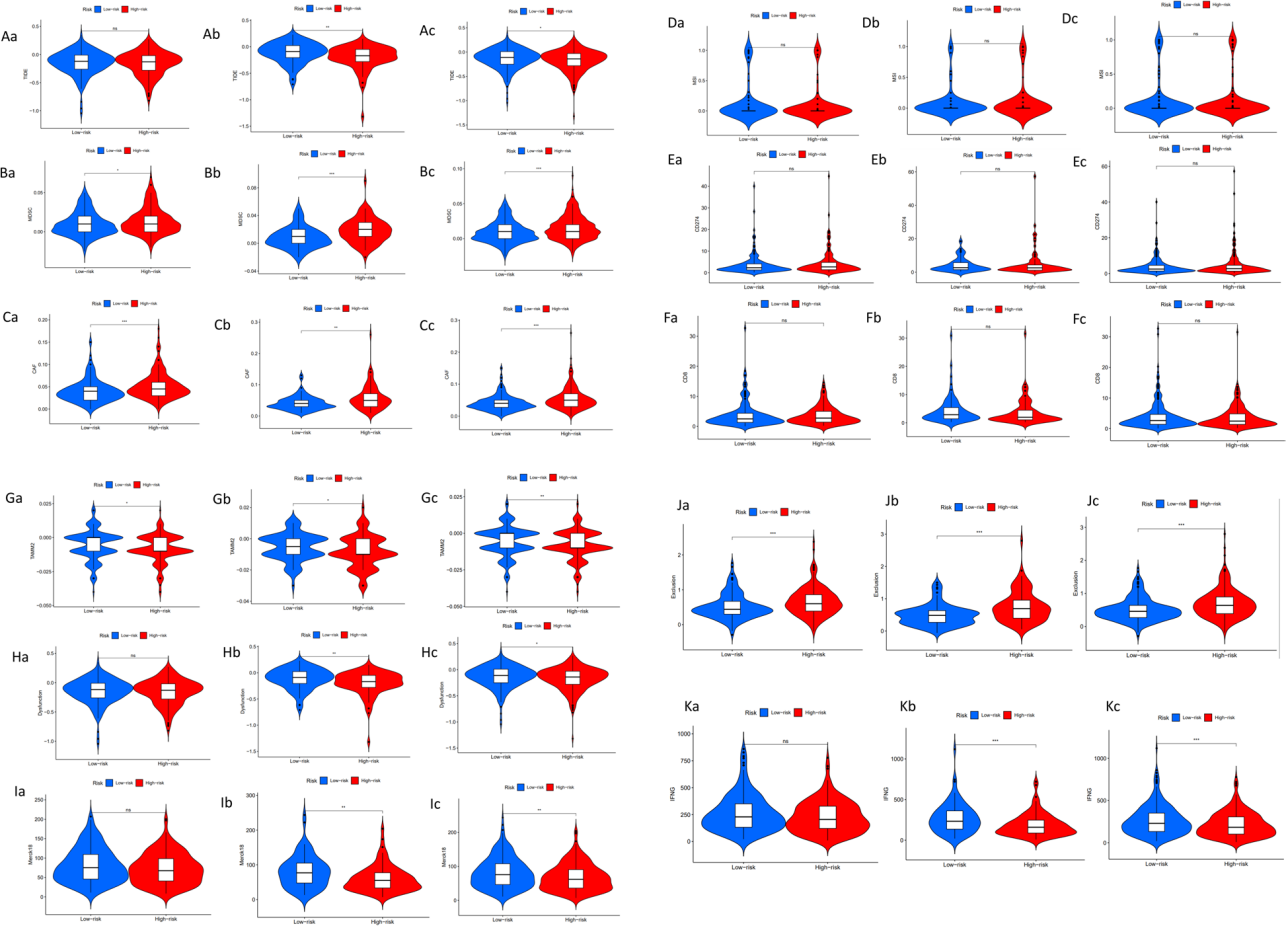
The differences of tumor markers in high-risk and low-risk subgroups. Among them, “_a” means in the training dataset, “_b” means in the testing dataset, and “_c” means in the entire dataset. “*” means that *p* < 0.05, “**” means that *p* < 0.01, “***” means that *p* < 0.001, “ns” means no significant

We conducted an analysis and visualization of tumor immune escape and immunotherapy for (CTLA-4)-related lncRNAs in high-risk and low-risk subgroups. Figure 4 illustrates that the TIDE scores of the testing dataset (*p* < 0.01, **) and entire datasets (*p* < 0.05, *) were lower in the high-risk subgroup compared to the low-risk subgroup, indicating that lower TIDE scores correspond to higher risk scores, which is an unexpected finding (Fig. 4A b-c). However, the result in the training dataset was not significant (Fig. 4A a). Across the training, testing, and entire datasets, there was a notable increase in the presence of MDSC and CAF within the high-risk subgroup as compared to the low-risk subgroup. This suggests a correlation wherein higher levels of MDSC (Fig. 4B a-c) and CAF (Fig. 4C a-c) align with elevated risk scores. Other immune markers, such as MSI (Fig. 4D a-c), CD274 (Fig. 4E a-c), CD8 (Fig. 4F a-c), TAM M2 (Fig. 4G a-c), immune dysfunction (Fig. 4H a-c), Merck 18 (Fig. 4I a-c), immune exclusion (Fig. 4J a-c) and IFNG (Fig. 4K a-c), are also presented in the figures.

#### Drug selection for CTLA4LncSigs

To pinpoint potential chemotherapy drugs for CTLA4LncSigs, we employed the "pRRophetic" package with R software to assess drug responses within both high-risk and low-risk subgroups across the training, testing, and entire datasets. We identified 67 drugs in the training dataset, 59 in the testing dataset, and 77 in the entire dataset with significantly different estimated IC50 values between the high-risk and low-risk subgroups. Most drugs exhibited lower IC50 values in the high-risk subgroup. A subset of these drugs is presented in Figure S7A-L. IC50 of Erlotinib is lower in high-risk group than in low-risk group (Figure S7I). We inferred that it is related to FAM66C, one of the CTLA4LncSigs. FAM66C has a negative correlation (HR > 1) which means it is a risk factor for LUAD. High expression of FAM66C activates EGFR-ERK signaling by inhibition of the proteasome pathway promoting tumor growth [43]. Erlotinib is a specific inhibitor that targets EGFR and inhibits EGFR phosphorylation to block EGFR-ERK signaling [44]. Therefore, the IC50 of Erlotinib is lower in the high-risk group. Conversely, only a few drugs, such as BI.2536 and BIRB.0796 in the training dataset (Figure S7M-N), Methotrexate in the testing dataset (Figure S7O), and Lenalidomide, Methotrexate, and PD.0332991 in the entire dataset (Figure S7P-R), exhibited greater sensitivity in the low-risk subgroup. These indicate that the risk score is strongly associated with IC50 so we can apply different drugs according the risk score, thereby supporting personalized treatment strategies. We suggest future research directions to validate these findings in larger, independent cohorts and experimental studies. This could include functional assays to elucidate the role of CTLA-4-related lncRNAs in mediating drug sensitivity and resistance.

#### Causal effects of nitrogen metabolism on lung cancer

**Fig. 5:**
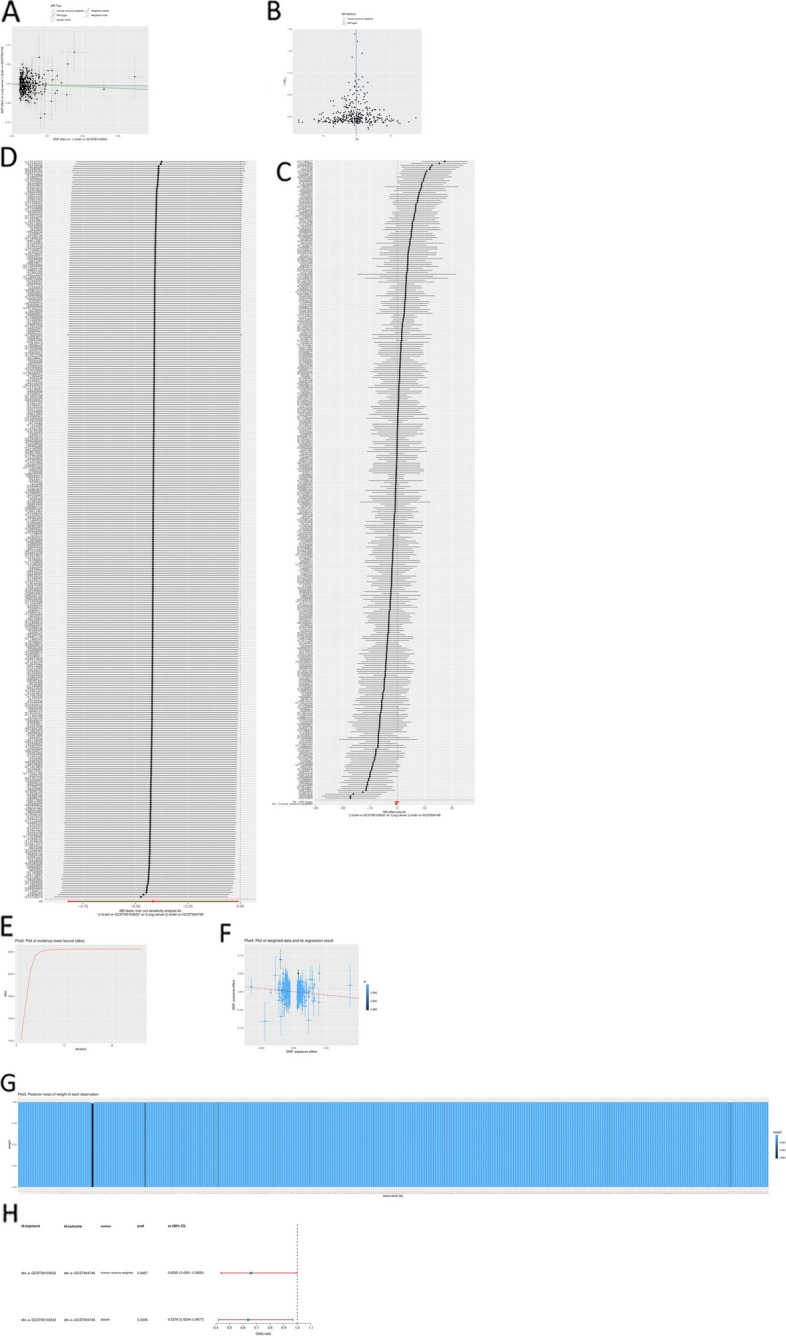
The illustration of the MR Method. **A**, **B** further demonstrated that the 2SMR analysis results were dependable. The effects of each SNP on lung cancer are shown in (**C**). The results of LOO MR (**D**). The ELBO curve (**E**). The observations are consistent with the hypothesis of the instrumental variables, and the estimation of the causal effect is reliable **F**. The posterior mean of the weight of each observation is close to 1, indicating that these observations contribute highly to the estimate of causal effects (**G**). The odds ratios of MR and BWMR (**H**)

The result of IVW indicated that blood urea nitrogen levels were significantly associated with lung cancer (*p* < 0.05) (Table S7). Meanwhile, the pleiotropic test showed no notable horizontal bias in 2SMR results (*p* > 0.05), which expressed that our 2SMR results were reliable (Table S6). The line direction of these methods is from top left to bottom right and roughly the same, which means that the nitrogen metabolic pathway is a protective factor for lung cancer (Fig. 5A). The number of black dots on both sides of the line is roughly the same, which indicates that the results obtained by the IVW method are reliable (Fig. 5B). Figure 5C also showed the combined effect of these SNPs on lung cancer is protective in the IVW method. The results of LOO MR (Fig. 5D) indicated that MR analysis was not affected by an individual SNP, that is, MR sensitivity analysis was qualified.

Evidence Lower Bound (ELBO) is an indicator used to measure the quality of the data fitted to a model. The ELBO tends to a constant, indicating that the model has converged, and the model has a high degree of fitting to the data, that is, there is a strong causal relationship between the model and lung cancer (Fig. 5E). A high weight indicates that the observations conform to the assumptions of the instrumental variables and that the estimates of causal effects are reliable. In Fig. 5G, almost all observations have a high weight, indicating that these observations contribute significantly to estimates of causal effects. Regression results of SNP-exposure and SNP-outcome effects for each observed value confirmed a significant association between nitrogen metabolism and lung cancer (Fig. 5F).

The odds ratio of the IVW method and BWMR analysis were both less than 1 (*p* < 0.05), suggesting that nitrogen metabolism was a protective factor for lung cancer (Fig. 5H). KEGG showed that nitrogen metabolic pathway includes nitrification. High doses of the nitration inhibitor Nitrapyrin cause liver tumors in mice [45, 46]. Overall, the results of MR Analysis showed a significant association between nitrogen metabolic pathways and lung cancer, indicating that our model is dependable.

## Discussion

In recent decades, lung cancer has remained a significant cause of morbidity and mortality. Present diagnostic techniques, including imaging and pathological biopsy, exhibit constraints in predicting the prognosis of cancer patients on a large scale. In this study, we initially identified CTLA4LncSigs associated with prognostic risk signatures. We observed the lncRNAs AP000253.1 and FAM66C displayed higher expression levels in the high-risk subgroup, indicating their association with increased tumor risk. It is worth noting that these lncRNAs have been found to play diverse roles in previous studies [47, 48]. For instance, FAM66C was found to inhibit the proliferation of pancreatic cancer cells [49], while paradoxically promoting the proliferation of prostate cancer cells [50]. Although no effect of high or low-risk scores on patient response to medication was observed, risk score models based on CTLA4LncSigs were superior to clinicopathological factors in predicting efficacy.

GO functional analysis revealed that the mainly enriched BP terms were cornification, epidermis development and aging. The mainly enriched CC terms included cell–cell junction and apical plasma membrane. MF terms mainly enriched extracellular matrix structural constituent. The favorable effect of cell–cell junction on the collective invasion of tumor cells has been demonstrated [30]. Past research has already established the connection between lung cancer and apical plasma membrane and aging [33]. Extracellular matrix structural constituent was associated with lung metastasis in colorectal cancer [34, 35]. Subsequently, KEGG enrichment pathway analysis revealed three significant enrichment pathways for amoebic infection, Staphylococcus aureus infection, and nitrogen metabolism. It has been observed that nitrogen metabolism in cancer tissues differs significantly from that in normal tissues. The demand for nitrogen is significantly increased in rapidly proliferating tumor cells. Nitrogen plays a key role in the malignant progression of cancer [37, 38].

The results of MR Analysis also confirmed that the activity of the nitrogen metabolism pathway is a protective factor for lung cancer. KEGG showed that nitrogen metabolic pathway includes nitrification. High doses of the nitration inhibitor Nitrapyrin cause liver tumors in mice [45, 46]. An active nitrogen metabolism pathway leads to an increase in glutamate production. Then accumulated glutamate enhances ferroptosis after the inhibition of system Xc-, and ultimately LUAD cells were sensitized to ferroptosis [51]. In addition, carbamoly-P in the nitrogen metabolism pathway is involved in arginine biosynthesis. Arginine is essential for T cell survival, proliferation and functional expression [52]. Elevated arginine levels affect T-cell activation, differentiation, and function, resulting in better antitumor activity [53–55]. At the same time, the active nitrogen metabolic pathway also promotes the transformation and excretion of ammonia. Ammonia, as a metabolic waste, not only induces T-cell failure but also promotes tumor growth [56]. Ammonia can also be taken up by some cancer cells as a biosynthetic metabolite to drive amino acid metabolism [57]. These discoveries imply a strong link between lncRNAs related to CTLA-4 and the onset and progression of cancer. The predictive ability of lncRNA deserves further study.

It is undeniable that alterations in the tumor microenvironment play a crucial role in cancer progression. We identified a cluster of immune-function pathways: check-point and T cell co-stimulation, which we show are linked to low risk of tumor. These pathways also play a similar role in genes associated with Solute carrier transporters (SLC) in osteosarcoma [58]. In patients with squamous cell carcinoma of the oral tongue, high immune cytolytic activity was associated with a better prognosis [40]. Parainflammation is a procarcinogenic inflammatory process which could be blocked by NSAIDs [42]. Patients characterized by both high TMB and low-risk scores exhibited a heightened survival rate compared to their counterparts, while MDSC and CAF were negatively associated with survival. Previous study has also demonstrated the involvement of MDSC and CAF in tumor immune evasion in hepatocellular carcinoma [59].

Despite the comprehensive evaluation of our models, their construction and validation relied on existing data from a publicly available database, which introduces certain limitations. It is imperative to conduct further external and practical testing to validate the precise predictive efficacy of our models for clinical patients.

In conclusion, this study provides valuable insight into the prognosis of patients with lung adenocarcinoma. We have a preliminary understanding of the correlation between CTLA-4 related lncRNAs and tumor progression and the mechanism. The effect of nitrogen metabolism on the tumor microenvironment of lung adenocarcinoma deserves in-depth study. The innovative prognostic model we developed based on CTLA-4-associated lncRNA has strong predictive power for the overall survival of LUAD patients. This has the potential to guide future diagnosis and treatment of LUAD.

### Supplementary Information

Supplementary Material 1

## Data Availability

All data generated or analyzed during this study are included in this published article. The datasets used and/or analyzed during the current study are available from the corresponding author upon reasonable request. We welcome legitimate requests for raw data.
