# Supplementary material for: Combination of transcriptome and Mendelian inheritance reveals novel prognostic biomarker of CTLA-4-related lncRNAs and protective role of nitrogen metabolism pathway in lung adenocarcinoma development
Source: BMC Cancer. 2024 Aug 14;24:1009. doi: 10.1186/s12885-024-12777-7 (PMC11323378; doi:10.1186/s12885-024-12777-7)
Supplement: Supplementary file 1 — Supplementary Material 1 [file 12885_2024_12777_MOESM1_ESM.pdf]

Supplementary materials

Combination of transcriptome and Mendelian Inheritance reveals novel prognostic biomarker of CTLA-4-related lncRNAs and protective role of nitrogen metabolism pathway in lung adenocarcinoma development

Abbreviations 2

Supplementary Figure S1 3

Supplementary Figure S2 5

Supplementary Figure S3 7

Supplementary Figure S4 10

Supplementary Figure S5 11

Supplementary Figure S6 13

Supplementary Figure S7 14

Supplementary Table S1 20

Supplementary Table S2 21

Supplementary Table S3 28

Supplementary Table S4 30

Supplementary Table S5 34

Supplementary Table S6 35

Supplementary Table S7 35

Abbreviations

AJCC: American Joint Committee on Cancer; AUC: area under curve; BP: biological process; BWMR: Bayesian weighted Mendelian randomization; CAF: cancer-associated fibroblasts; CC: cellular component; CTLA-4: cytotoxic T-lymphocyte-associated protein 4; CTLA4LncSigs: CTLA-4-related lncRNA-based gene signatures; ELBO: Evidence Lower Bound; FDR: false discovery rate; GO: gene ontology; HR: hazard ratio; ICI: immune checkpoint inhibitor; IVW: inverse-variance weighted; KEGG: Kyoto Encyclopedia of Genes and Genomes; LASSO: least absolute shrinkage and selection operator; LncRNA: Long non-coding RNA; LOO MR: Leave-One-Out Mendelian Randomization; LUAD: lung adenocarcinoma; M: distant metastasis; MDSC: Myeloid-derived suppressor cell; MF: molecular function ; N: regional lymph nodes; OS: overall survival; ROC: receiver operating characteristic; T: primary tumor; TCGA: The Cancer Genome Atlas; TMB: Tumor Mutation Burden; TME: tumor microenvironment; 2SMR: 2-sample Mendelian randomization.

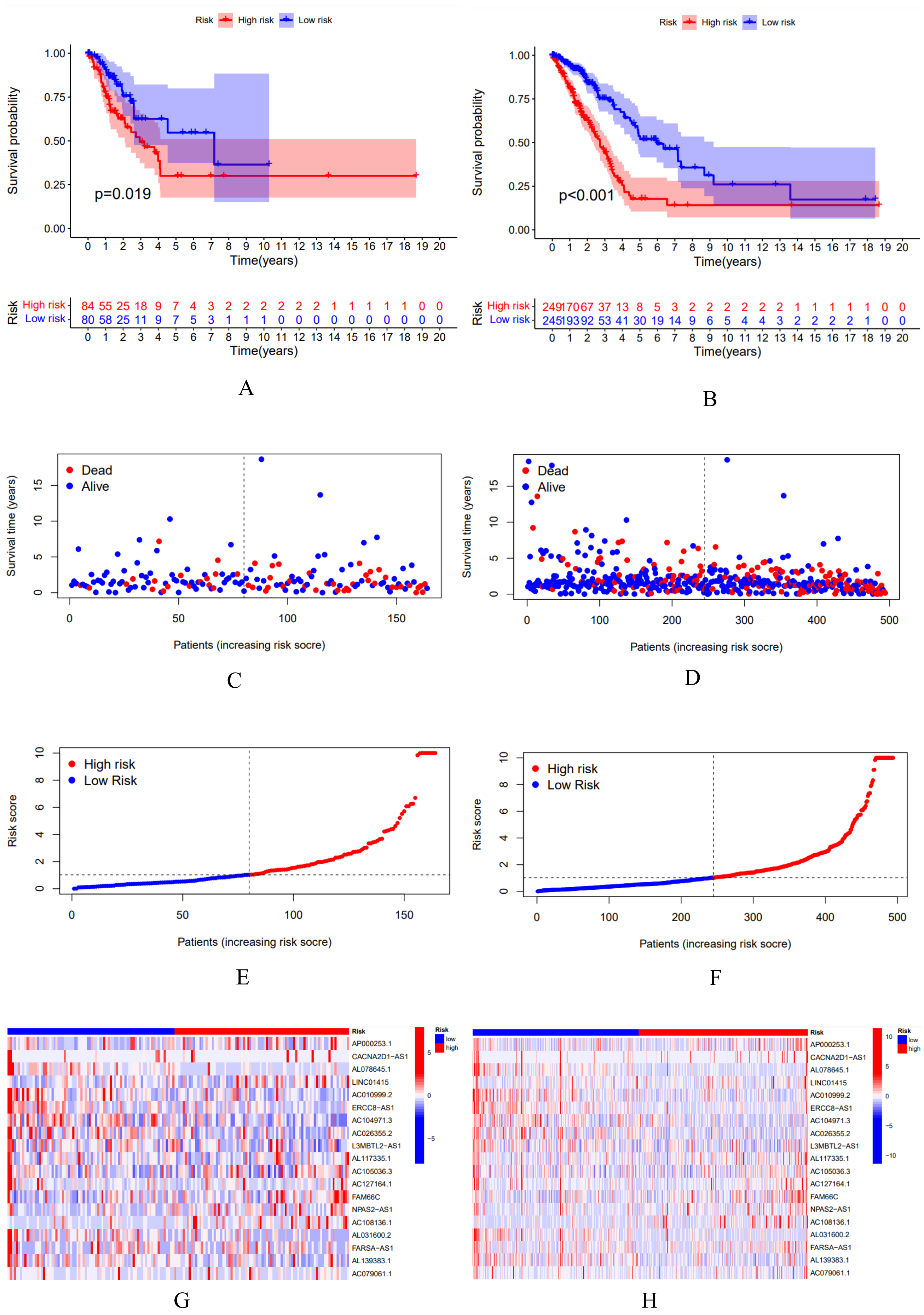
Risk
High risk
Low risk
Survival probability
p=0.019
p<0.001
Time(years)
A
B
Dead
Alive
Survival time (years)
Patients (increasing risk socre)
C
D
High risk
Low Risk
Risk score
E
F
AP000253.1
CACNA2D1-AS1
AL078645.1
LINC01415
AC010999.2
ERCC8-AS1
AC104971.3
AC026355.2
L3MBTL2-AS1
AL117335.1
AC105036.3
AC127164.1
FAM66C
NPAS2-AS1
AC108136.1
AL031600.2
FARSA-AS1
AL139383.1
AC079061.1
low
high
G
H

Testing Entire

Supplementary Fig. S1. Results of Kaplan-Meier method in testing and entire datasets.

The survival curves of testing (A) and entire datasets (B). (C) and (E) are the distribution of risk scores, and survival status in the testing dataset, respectively. (D), (F) are the distribution of risk scores, and survival status in the entire cohort, respectively. The expression of CTLA4LncSigs in testing (G) and entire datasets (H).

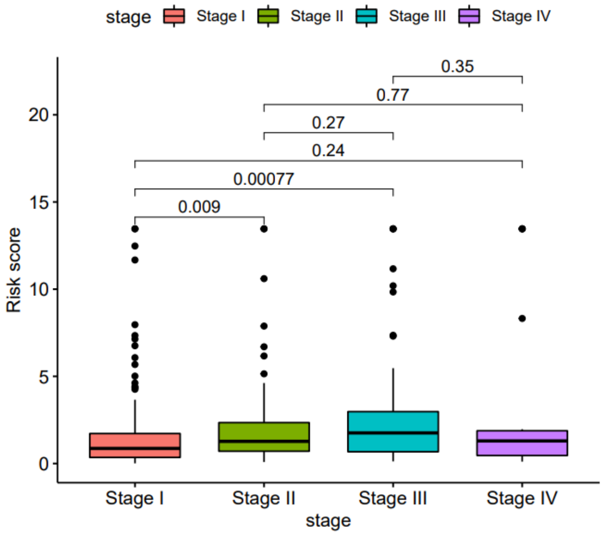
stage
Stage I
Stage II
Stage III
Stage IV
0.35
0.77
0.27
0.24
0.00077
0.009
Risk score
0
5
10
15
20
Stage I
Stage II
Stage III
Stage IV
stage

A

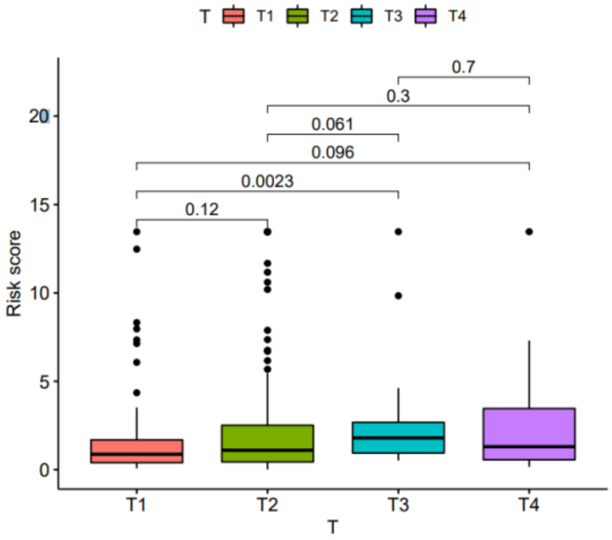
T
T1
T2
T3
T4
0.7
0.3
0.061
0.096
0.0023
0.12
Risk score
0
5
10
15
20
T1
T2
T3
T4
T

B

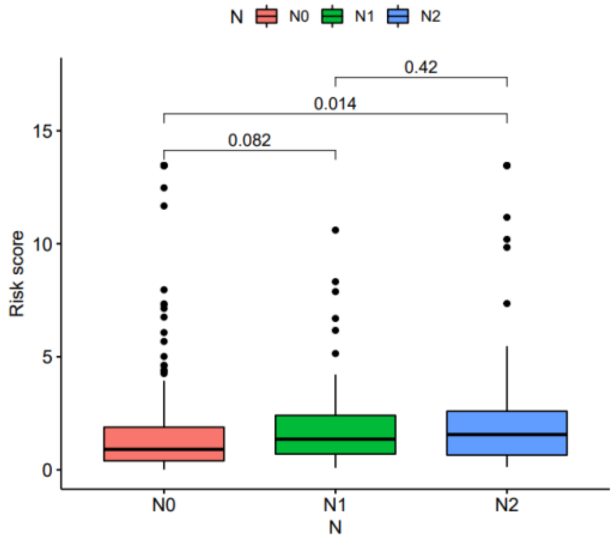
N
N0
N1
N2
0.42
0.014
0.082
Risk score
0
5
10
15
N0
N1
N2
N

C

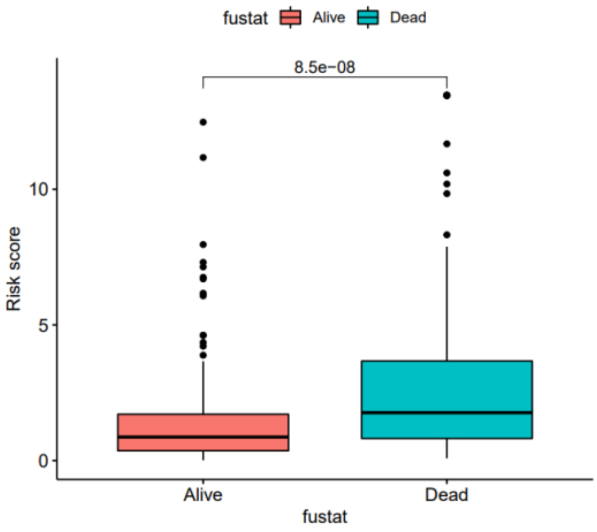
fustat
Alive
Dead
8.5e−08
Risk score
0
5
10
Alive
Dead
fustat

D

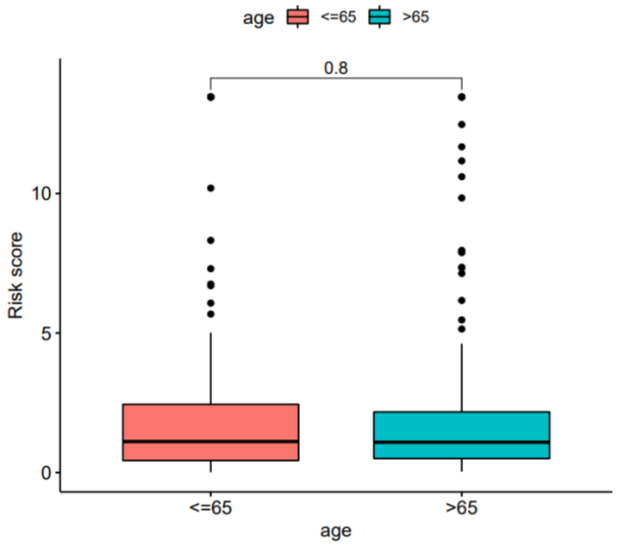
age
<=65
>65
0.8
Risk score
0
5
10
<=65
>65
age

E

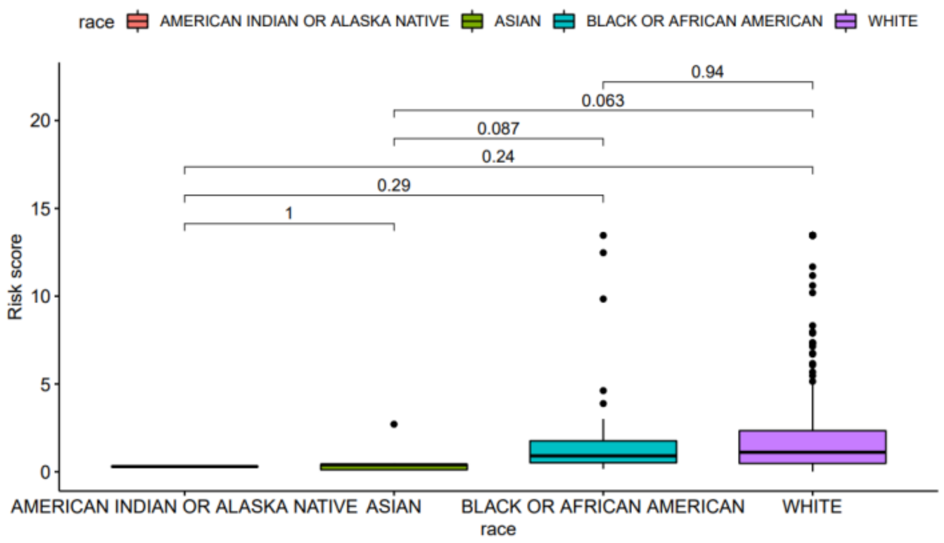

F

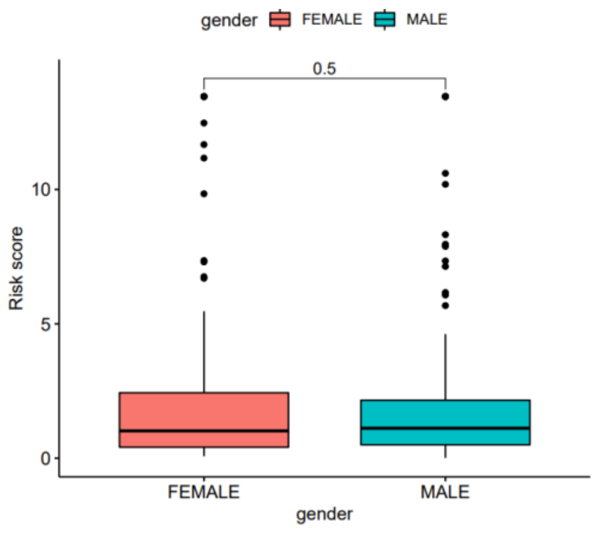

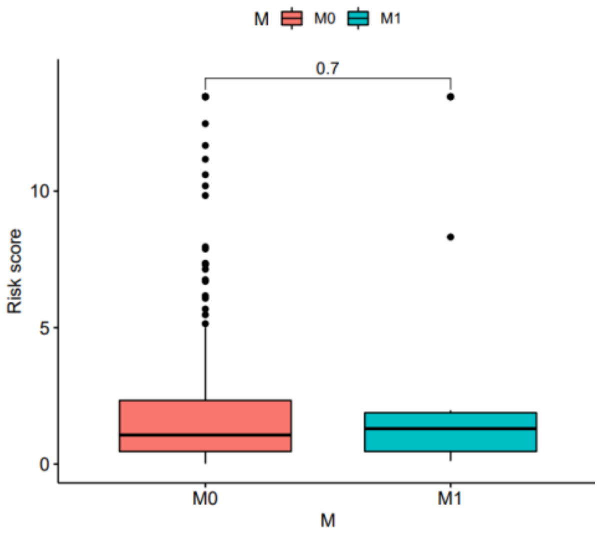

G H

Supplementary Fig. S2. The Risk Score of Patients Grouping by Different Clinicopathological Factors.

Which include AJCC stage (A), T stage (B), N stage (C), survival state (D), age (E), race (F), gender (G), and M stage (H).

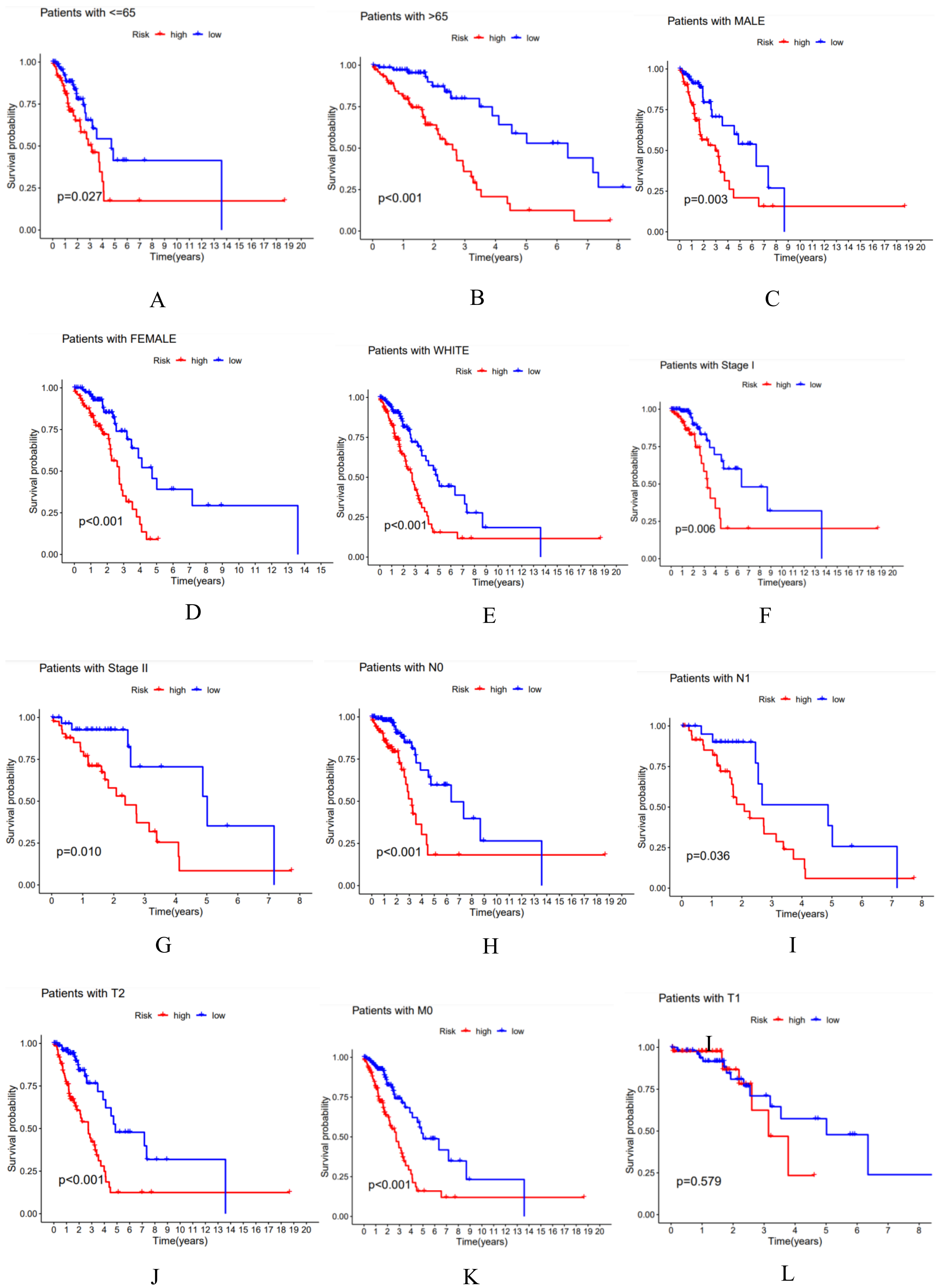
Patients with <=65
Risk high low
Survival probability
p=0.027
Time(years)
A
Patients with >65
Risk high low
Survival probability
p<0.001
Time(years)
B
Patients with MALE
Risk high low
Survival probability
p=0.003
Time(years)
C
Patients with FEMALE
Risk high low
Survival probability
p<0.001
Time(years)
D
Patients with WHITE
Risk high low
Survival probability
p<0.001
Time(years)
E
Patients with Stage I
Risk high low
Survival probability
p=0.006
Time(years)
F
Patients with Stage II
Risk high low
Survival probability
p=0.010
Time(years)
G
Patients with N0
Risk high low
Survival probability
p<0.001
Time(years)
H
Patients with N1
Risk high low
Survival probability
p=0.036
Time(years)
I
Patients with T2
Risk high low
Survival probability
p<0.001
Time(years)
J
Patients with M0
Risk high low
Survival probability
p<0.001
Time(years)
K
Patients with T1
Risk high low
Survival probability
p=0.579
Time(years)
L

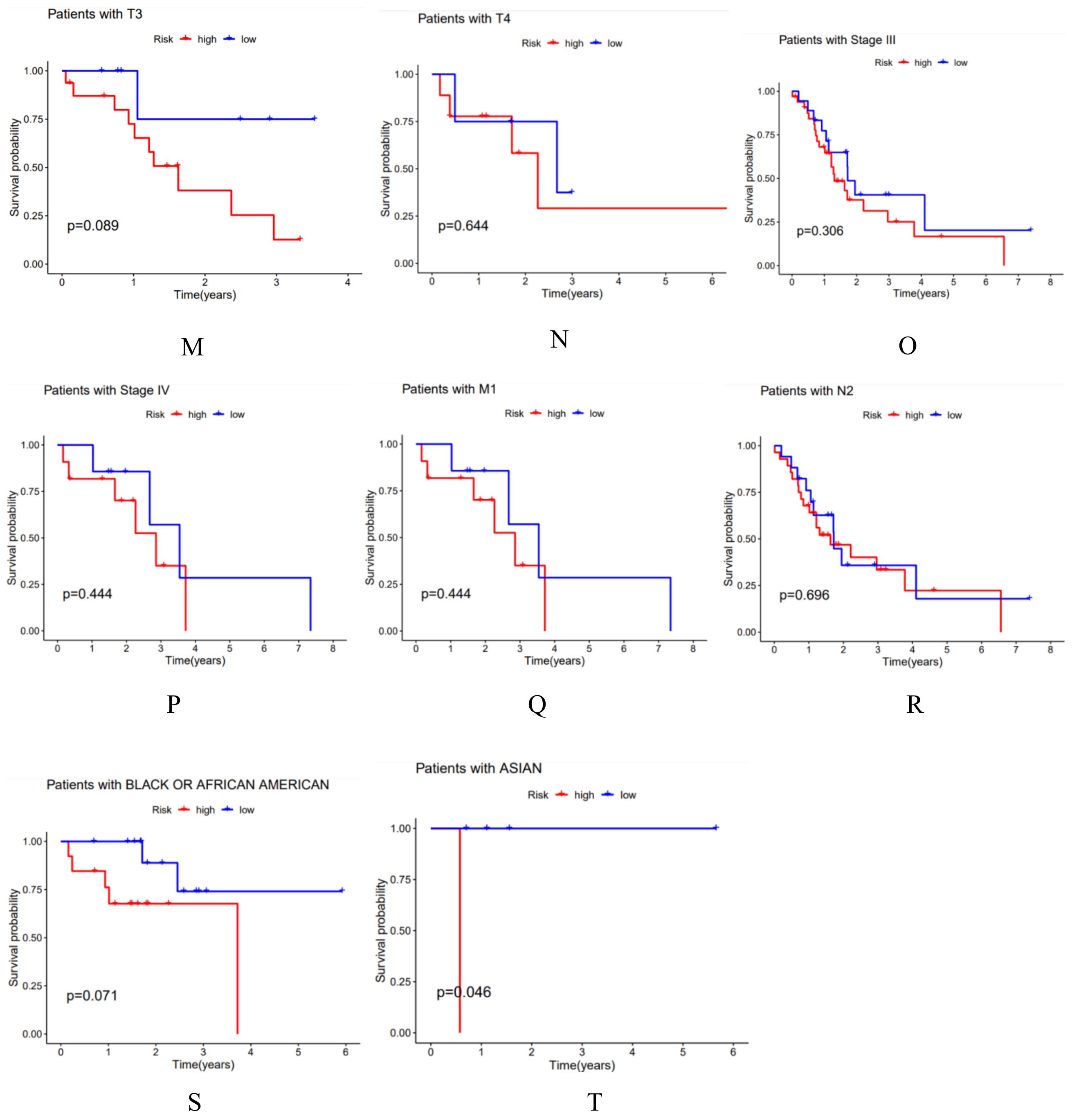

Supplementary Fig. S3. Overall Survival of Patients in High-risk and Low-risk Subgroups with Different Clinicopathological Factors.

The p value less than 0.05 were age ≤ 65 (A) and > 65 (B), male (C) and female (D), race-white (E), AJCC stages I-II (F-G), N0-N1 stage (H-I), T2 stage (J), M0 stage (K) and race-Asian (T). Overall Survival of Patients in High-risk and Low-risk Subgroups with Different Clinicopathological Factors

($p \geq 0.05$). Which include T1 (L) and T3-T4 stage (M-N), AJCC stages III-IV (O-P), M1 stage (Q), N2 stage (R), race-black or African American (S).

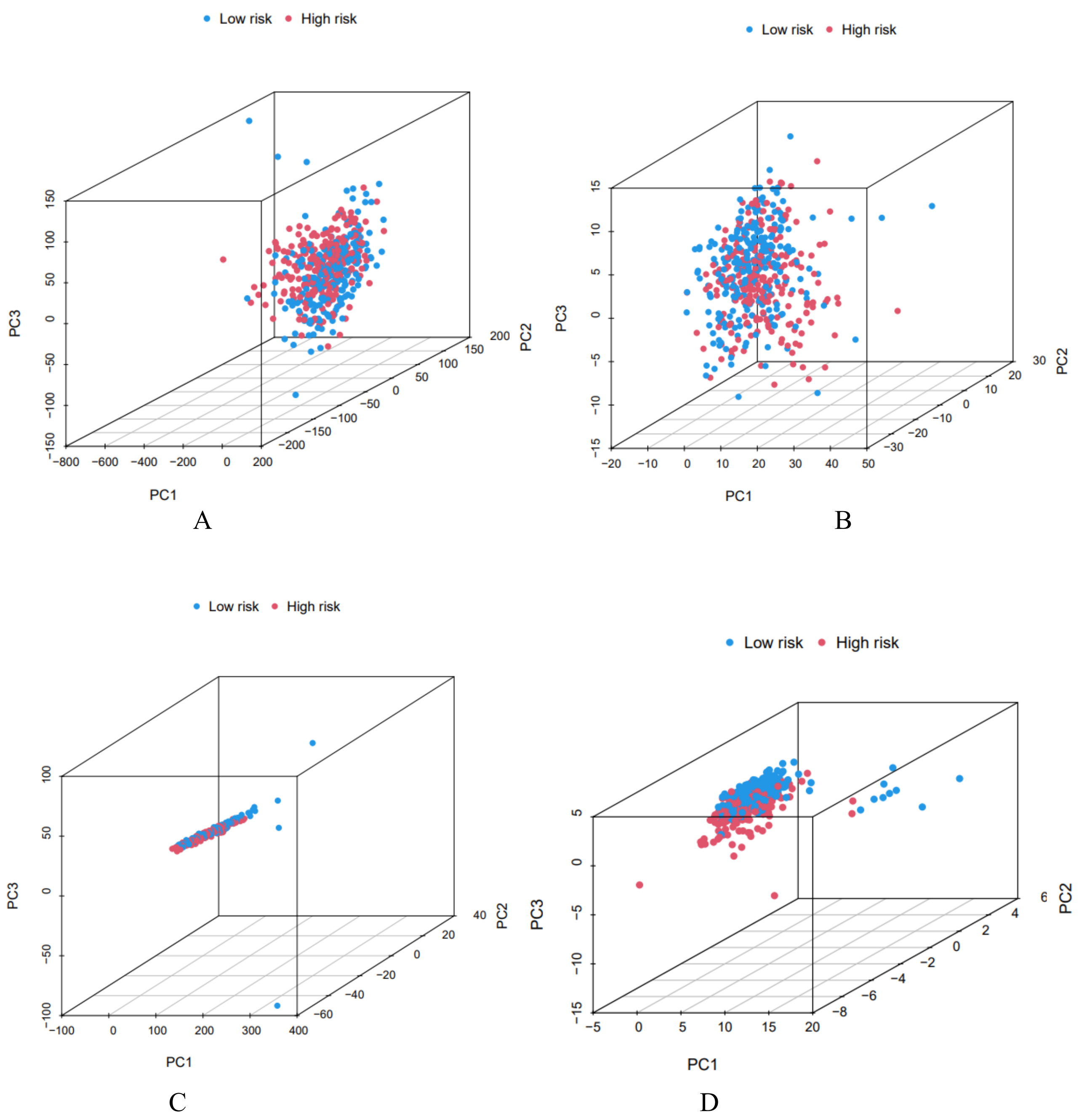

Supplementary Fig. S4. The 3D images of PCA results.

(A) is LUAD’s genes. (B) is LUAD’s mRNAs. (C) is LUAD’s lncRNAs. (D) is 19CTLA4LncSigs.

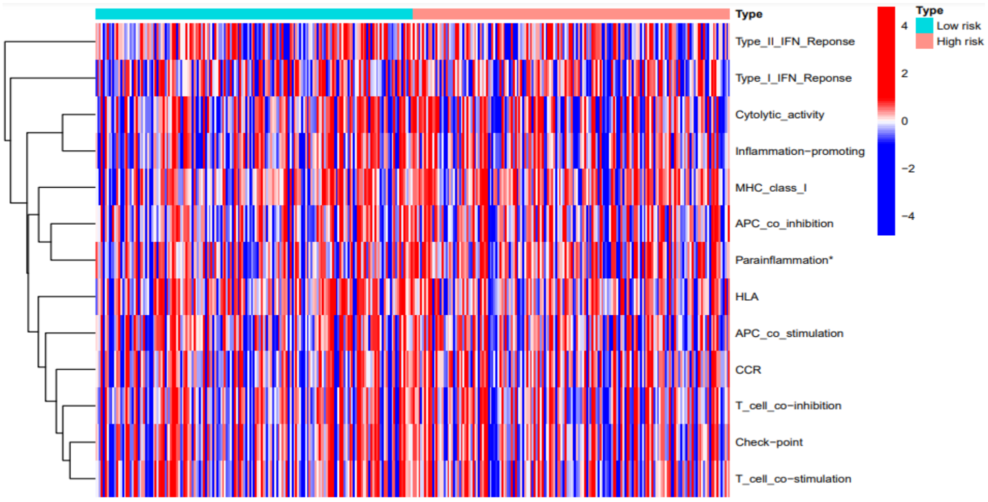

A

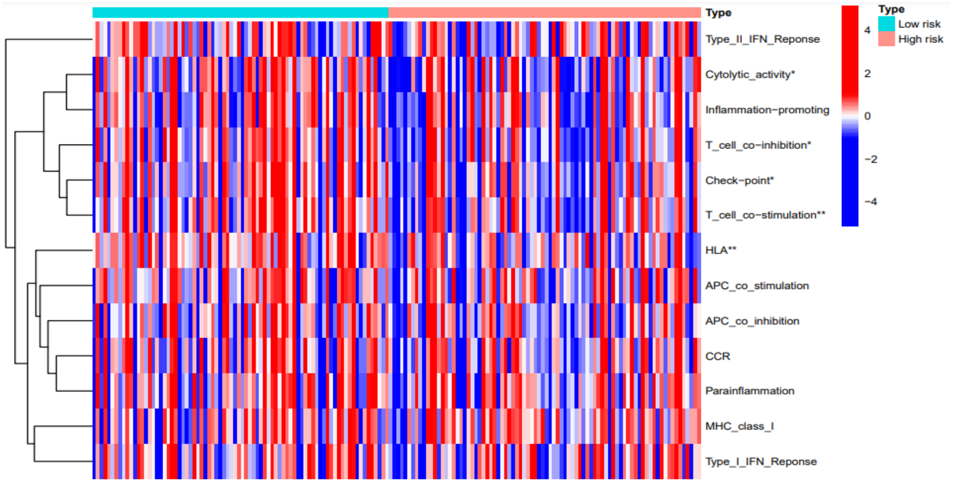

B

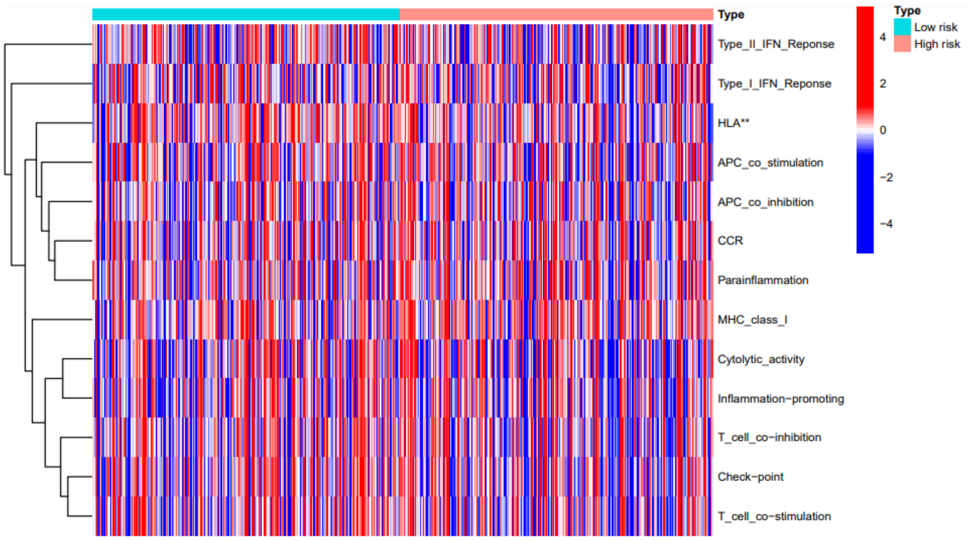

C

Supplementary Fig. S5. The heat map of the expression of 13 immune functions between

high-risk and low-risk subgroups. In the training dataset (A). In the testing dataset (B). In the entire dataset (C).

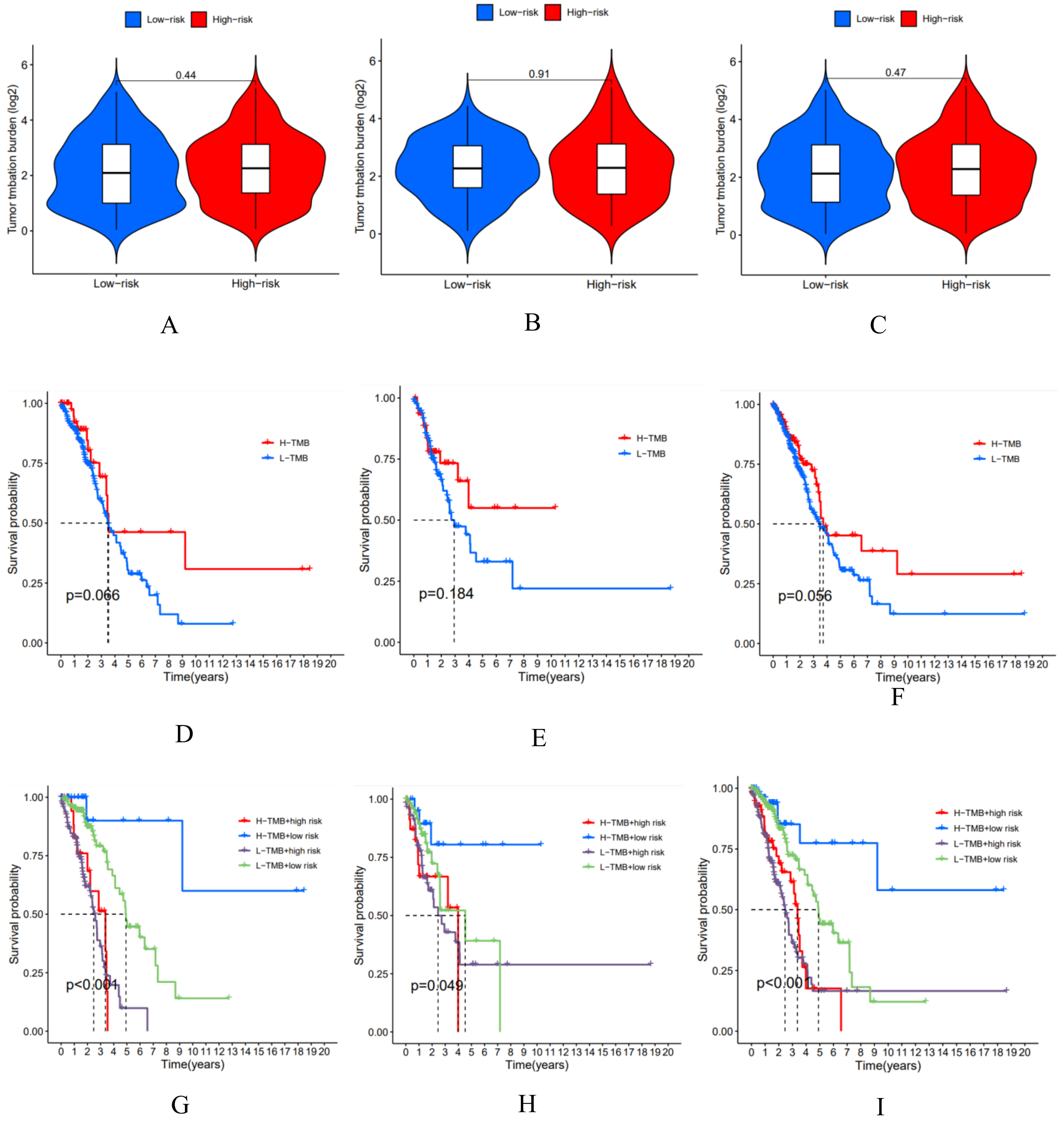

Supplementary Fig. S6. The effect of TMB on OS of LUAD patients.

TMB between high-risk and low-risk subgroups in training (A), testing (B), and entire datasets (C). The relationship between TMB and OS of LUAD patients in training (D), testing (E), and entire dataset (F). The impact of TMB combined with risk score on the OS of LUAD patients in training (G), testing (H), and entire datasets (I).

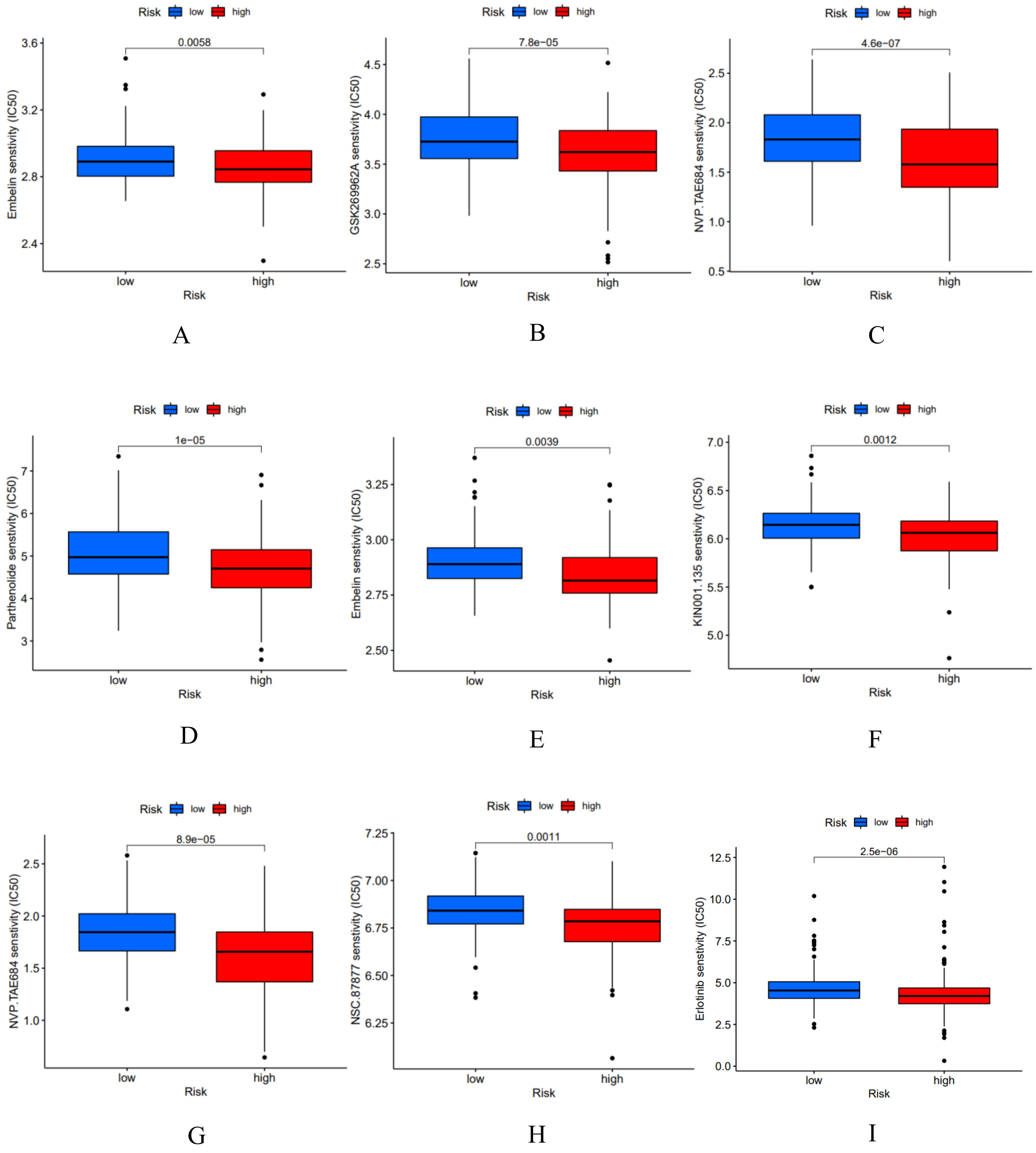

Risk low high
0.0058
Embelin senstivity (IC50)
Risk
A
7.8e−05
GSK269962A senstivity (IC50)
B
4.6e−07
NVP.TAE684 senstivity (IC50)
C
1e−05
Parthenolide senstivity (IC50)
D
0.0039
Embelin senstivity (IC50)
E
0.0012
KIN001.135 senstivity (IC50)
F
8.9e−05
NVP.TAE684 senstivity (IC50)
G
0.0011
NSC.87877 senstivity (IC50)
H
2.5e−06
Erlotinib senstivity (IC50)
I

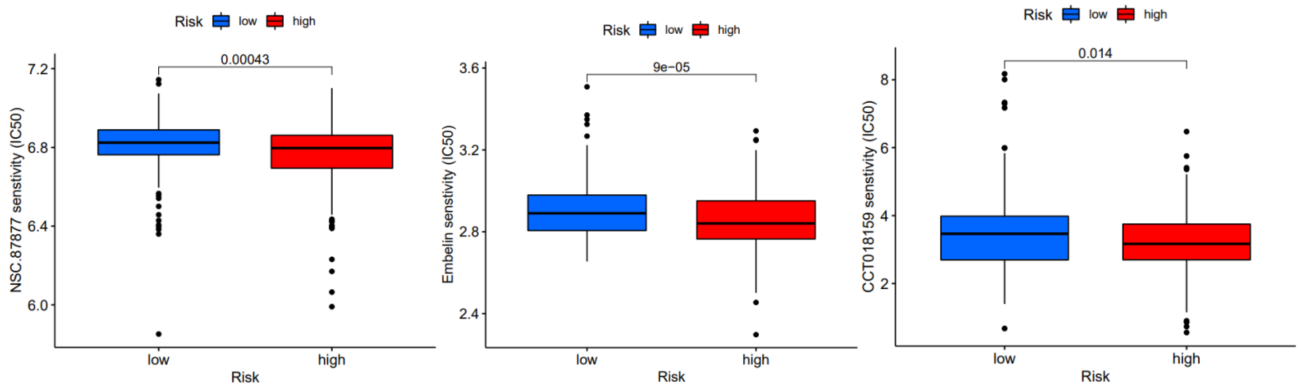

J K L

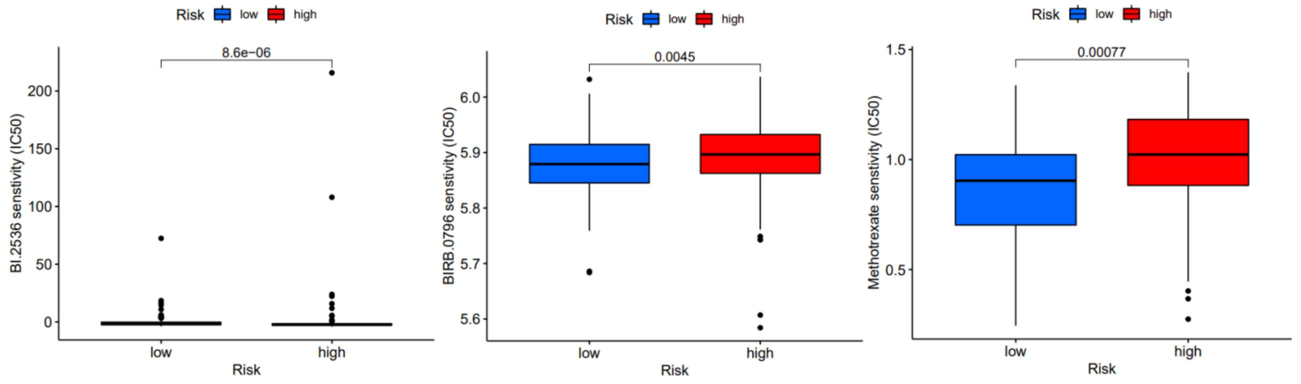

M N O

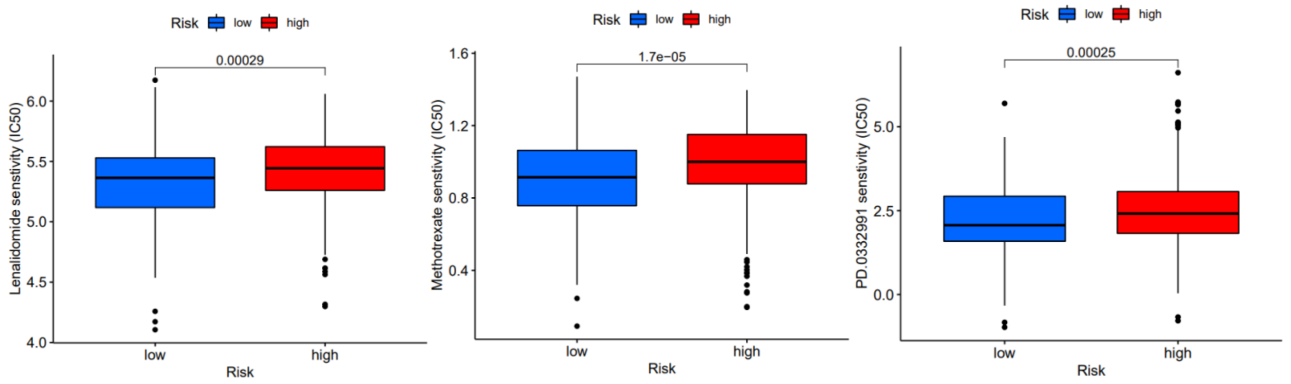

P Q R

Training Testing Entire

Supplementary Fig. S7. The IC50 of the drugs in different subgroups.

(A) to (L) are the drugs that are more sensitive to the high-risk subgroup (partial). (A), (B), (C) and (D) are in the training dataset, (E), (F), (G), and (H) are in the testing dataset, (I), (J), (K) and (L) are in the entire dataset. (M) to (R) are drugs that are more sensitive to the low-risk subgroup. (M) and

(N) were in the training dataset, and (O) was in the testing dataset. (P), (Q) and (R) were in the entire dataset.

Supplementary Table S1. Details of Blood urea nitrogen levels and Lung cancer.

Trait	PMID	Year	Population	Sample size	Number of SNPs	Author
**Blood urea nitrogen levels**	34272381	2021	——	173,149	7,777,636	Stanzick KJ
**Lung cancer**	28604730	2017	European	85,716	7,857,154	McKay JD

Supplementary Table S2. Results of the univariate Cox regression analysis of the entire dataset.

gene	HR	HR.95L	HR.95H	p-value
**AC004080.2**	1.082934336	1.032151923	1.13621527	0.00114846
**NR2F2-AS1**	1.306599356	1.048744571	1.627852885	0.017111935
**AC007728.2**	0.786468106	0.620938674	0.996124267	0.046352346
**AC008073.2**	0.407311154	0.169054945	0.981351808	0.045293251
**AC064875.1**	1.587872928	1.00553295	2.507466748	0.047296609
**AC090236.2**	0.662104917	0.449126354	0.976079263	0.037321518
**AC099788.1**	1.34695439	1.027329144	1.766022252	0.03115802
**AC011586.2**	1.261872188	1.108109735	1.436970877	0.00045086
**AF131215.6**	0.887368506	0.800090567	0.984167165	0.023692245
**LUADT1**	1.196861163	1.000992956	1.431055668	0.048741194
**AL135960.1**	0.233303923	0.06776693	0.803204761	0.021032821
**AL021026.1**	0.573753614	0.335903377	0.980023521	0.041968097
**LINC00996**	0.840768241	0.736831905	0.959365671	0.009991709
**AL136304.1**	0.905141689	0.831744178	0.985016185	0.020895598
**SH3BP5-AS1**	0.951126208	0.912470271	0.991419767	0.017931681
**AC012085.2**	1.037142441	1.012903986	1.061960914	0.002505953
**AP000302.1**	0.380789585	0.159933577	0.906630807	0.029151933
**AC018529.1**	0.63885936	0.451407665	0.904152308	0.01145316
**AL031667.3**	1.055275844	1.016132605	1.09592695	0.00527382

**AC006017.1**	0.851433952	0.740034021	0.979603308	0.024576173
**AC015914.1**	0.793748458	0.633643571	0.994307594	0.044470351
**AC087501.4**	0.796909029	0.656399381	0.967496343	0.021799089
**AC087854.1**	0.691122933	0.485657119	0.983514687	0.040139858
**AC025279.1**	0.198285619	0.043588409	0.902010141	0.036314309
**AL353801.3**	0.731647996	0.559237865	0.957211276	0.022671792
**ABALON**	1.118352717	1.032462494	1.21138812	0.006078346
**AC073316.1**	1.135614569	1.004062564	1.284402483	0.04291815
**LINC01537**	1.306531555	1.184129093	1.441586661	9.96E-08
**LINC01415**	1.373092184	1.125922437	1.674522227	0.001740627
**AC083967.1**	1.046358692	1.022015394	1.071281822	0.000161214
**AP001178.1**	1.263705069	1.004861171	1.589225007	0.045345748
**AL049775.1**	1.190728633	1.032466617	1.373249899	0.016437001
**AC012050.1**	1.207254856	1.069214997	1.363116202	0.002364009
**PAN3-AS1**	0.847303211	0.722325745	0.993904393	0.041845007
**FAM30A**	0.916719953	0.844818444	0.994740916	0.036934112
**AL109811.1**	0.512169913	0.28061647	0.934791959	0.029285274
**AC010999.2**	0.562749926	0.367079407	0.862721998	0.008355905
**AC021086.1**	2.481531523	1.192793485	5.162669627	0.015030732
**TMPO-AS1**	1.099220753	1.050127106	1.150609539	4.95E-05
**LINC02587**	1.01053917	1.00416145	1.016957397	0.001172202
**PRR34**	1.467169103	1.027510304	2.0949524	0.034919188

**AC090559.1**	0.941808337	0.898195661	0.987538664	0.013200298
**ATP13A4-AS1**	0.946756479	0.903615246	0.991957401	0.021486819
**LINC00519**	1.045403901	1.022542401	1.068776527	8.29E-05
**IL12A-AS1**	1.099924253	1.038743831	1.164708107	0.001107114
**AF131215.5**	0.911574537	0.840305868	0.9888877	0.025813824
**AC009318.2**	1.082688727	1.015125777	1.154748412	0.015665776
**ADPGK-AS1**	0.297316148	0.109276019	0.80893221	0.017540705
**AC104971.3**	0.86334478	0.750256663	0.993478959	0.040238158
**MIR4527HG**	1.121364663	1.05771455	1.188845051	0.000122069
**LINC00626**	1.113982574	1.026782333	1.208588361	0.009445927
**AC092171.2**	1.017951948	1.002707883	1.033427767	0.020819764
**LINC01876**	1.055222773	1.007461876	1.105247878	0.022933194
**AC009318.3**	1.106497465	1.016513364	1.204447165	0.019365336
**AC099524.1**	0.72835673	0.539526605	0.983275933	0.038441712
**AC005291.1**	1.039277216	1.011435646	1.067885174	0.005424553
**AC026355.2**	0.916802154	0.870389078	0.965690184	0.001048816
**L3MBTL2-AS1**	0.770753714	0.610807399	0.972583646	0.028219451
**AC026310.2**	0.295973674	0.08774283	0.99837692	0.049694705
**AC090948.1**	0.865076447	0.762808529	0.98105518	0.023950003
**HLA-DQB1-AS1**	0.975567788	0.961741079	0.989593281	0.000682884
**AL121820.1**	1.158074104	1.056123853	1.269865864	0.001800263
**SMILR**	1.045403997	1.025164542	1.066043031	8.53E-06

**AL117335.1**	1.302721333	1.063943904	1.595086793	0.010468278
**AC105036.3**	1.269835586	1.027776973	1.568903038	0.026837373
**AC079949.1**	1.010203792	1.000286106	1.020219812	0.043717118
**AL606834.1**	1.057295742	1.004128165	1.113278489	0.034305671
**AC108134.4**	0.931326444	0.867690202	0.999629758	0.048813283
**HAGLR**	0.994663907	0.989501019	0.999853734	0.043898925
**AL359878.1**	0.712798689	0.521658622	0.973974071	0.033542924
**FAM66C**	1.511295301	1.140214763	2.003143234	0.004069069
**LINC01800**	0.159773076	0.044083746	0.57906685	0.005245642
**AC025419.1**	1.022107018	1.012496922	1.031808328	5.71E-06
**AL359232.1**	1.66091549	1.050364242	2.626365363	0.029996673
**AL021368.2**	0.761648876	0.580252637	0.999752476	0.049791871
**FOCAD-AS1**	0.666813592	0.450880406	0.986160324	0.042380174
**LINC00598**	1.282973887	1.11474256	1.476593838	0.000511547
**AP003064.2**	0.795293091	0.642010146	0.98517306	0.036019406
**LINC02360**	1.046899739	1.010074356	1.085067706	0.012120729
**AC012676.4**	0.661909175	0.455714996	0.961398591	0.030259806
**AC108136.1**	1.073737122	1.04254361	1.105863961	2.25E-06
**AL360270.1**	1.136150535	1.007353352	1.281415342	0.037588916
**LINC01150**	0.863347547	0.766981071	0.97182188	0.014962121
**LINC01385**	1.081698859	1.029928338	1.136071686	0.001698365
**AC034102.8**	0.75289254	0.602980774	0.940075043	0.012229738

**LINC01600**	1.457417177	1.057064035	2.009400337	0.021526096
**AL121985.1**	0.455818791	0.207952639	0.999125431	0.049745124
**AP001528.1**	1.097766237	1.018284893	1.183451429	0.014995369
**AL356608.1**	0.255017943	0.070854663	0.917852802	0.036515016
**AC114550.2**	0.537777959	0.290685719	0.994906576	0.048128451
**AC103808.6**	1.156912255	1.026888805	1.303399121	0.016567364
**LINC00892**	0.75146881	0.607346068	0.929791766	0.008538081
**ABCA9-AS1**	1.567709086	1.283962628	1.914161458	1.02E-05
**AC107959.1**	0.765476361	0.594108125	0.986275115	0.038749139
**LINC02410**	0.509924384	0.260126444	0.99960186	0.049864612
**AC008764.2**	0.96430039	0.932004169	0.997715754	0.036479816
**AC010998.1**	0.762926945	0.584942734	0.995067533	0.045885401
**AL031600.2**	0.450346933	0.253046479	0.801482642	0.006680274
**AL162632.3**	3.695901635	1.69840779	8.042643809	0.000983583
**GLCCI1-DT**	0.661247844	0.447525873	0.977035604	0.037838445
**AC087752.3**	0.849131091	0.766026139	0.941251966	0.001857739
**AC103591.3**	0.947287851	0.897790108	0.999514546	0.047962833
**AL138789.1**	1.038011263	1.013629782	1.062979207	0.002096051
**AP000977.1**	0.291992556	0.092806311	0.918683784	0.035292162
**AC079061.1**	1.63700646	1.131837759	2.367645122	0.008852105
**AC046143.1**	1.1011228	1.042017881	1.163580245	0.000621294
**AC026356.1**	1.066578976	1.003872934	1.133201895	0.037069134

**LINC01747**	2.277931742	1.366151381	3.798241611	0.001599421
**CYP1B1-AS1**	0.750027118	0.563999287	0.997413811	0.047954784
**LINC00671**	0.322219065	0.106249596	0.977181372	0.045420097
**AP001094.3**	1.225739043	1.005455273	1.494284471	0.044031609
**DIRC3**	1.117901687	1.054523193	1.185089329	0.000182015
**AC090617.5**	0.907419292	0.8395815	0.980738346	0.014263096
**AC090023.2**	1.123323834	1.063139905	1.186914751	3.48E-05
**TSPOAP1-AS1**	0.720639205	0.54757979	0.948393043	0.019381398
**AC007613.1**	0.610590436	0.387570077	0.961943923	0.03339778
**LINC02728**	0.625632803	0.396747439	0.986563154	0.04357275
**AL358115.1**	1.299719611	1.058978439	1.595189293	0.01213272
**AF121898.1**	1.138634374	1.059795219	1.223338448	0.000390672
**ZEB2-AS1**	0.445124159	0.206371132	0.960093184	0.039036309
**AC005291.2**	1.021318746	1.00887915	1.033911724	0.000741416
**LINC02310**	1.197449903	1.079869984	1.327832324	0.000632815
**FRMD6-AS1**	1.584761118	1.250944422	2.007657379	0.00013606
**AL137779.1**	0.649380947	0.441341573	0.955485819	0.028447772
**AP000695.1**	1.064896756	1.027451617	1.103706572	0.00057579
**LINC02147**	0.100883916	0.021672901	0.469598633	0.003463647
**AC007686.2**	0.242396846	0.075328008	0.780005111	0.017471814
**AC091057.1**	1.121974531	1.021803951	1.23196514	0.015864765
**AC026368.1**	1.039398709	1.013263202	1.066208339	0.002939186

**LINC00377**	1.322247484	1.106745719	1.57971102	0.002088727
**ZFHX4-AS1**	1.215366094	1.052344445	1.403641887	0.007947861

Supplementary Table S3. Clinicopathological factors of lung adenocarcinoma between training and testing datasets.

Covariates	Type	Total	Test	Train	P-value
**fustat**	Alive	190(64.63%)	59(62.11%)	131(65.83%)	0.6212
**fustat**	Dead	104(35.37%)	36(37.89%)	68(34.17%)	
**age**	<=65	147(50%)	48(50.53%)	99(49.75%)	1
**age**	>65	147(50%)	47(49.47%)	100(50.25%)	
**gender**	FEMALE	155(52.72%)	45(47.37%)	110(55.28%)	0.2521
**gender**	MALE	139(47.28%)	50(52.63%)	89(44.72%)	
**race**	AMERICAN INDIAN OR ALASKA NATIVE	1(0.34%)	0(0%)	1(0.5%)	0.2494
**race**	ASIAN	5(1.7%)	1(1.05%)	4(2.01%)	
**race**	BLACK OR AFRICAN AMERICAN	27(9.18%)	13(13.68%)	14(7.04%)	
**race**	WHITE	261(88.78%)	81(85.26%)	180(90.45%)	
**stage**	Stage I	153(52.04%)	50(52.63%)	103(51.76%)	0.2399
**stage**	Stage II	72(24.49%)	26(27.37%)	46(23.12%)	
**stage**	Stage III	51(17.35%)	17(17.89%)	34(17.09%)	
**stage**	Stage IV	18(6.12%)	2(2.11%)	16(8.04%)	
**T**	T1	96(32.65%)	31(32.63%)	65(32.66%)	0.2763
**T**	T2	162(55.1%)	55(57.89%)	107(53.77%)	
**T**	T3	23(7.82%)	8(8.42%)	15(7.54%)	

**T**	T4	13(4.42%)	1(1.05%)	12(6.03%)	
**M**	M0	276(93.88%)	93(97.89%)	183(91.96%)	0.0845
**M**	M1	18(6.12%)	2(2.11%)	16(8.04%)	
**N**	N0	189(64.29%)	57(60%)	132(66.33%)	0.5663
**N**	N1	60(20.41%)	22(23.16%)	38(19.1%)	
**N**	N2	45(15.31%)	16(16.84%)	29(14.57%)	

The p value of each clinicopathological factor is over 0.05 which means that there were no statistical differences in each clinicopathological factor between the training and testing datasets. Our grouping is reasonable.

Supplementary Table S4. Selecting the CTLA4LncSigs by univariate Cox regression analysis in the risk score model.

id	HR	HR.95L	HR.95H	p-value
**NR2F2-AS1**	1.750195139	1.113988336	2.749744253	0.015171044
**AP000253.1**	4.36550617	1.588968564	11.99372005	0.004262793
**AL137782.1**	1.399030806	1.042320215	1.877817554	0.025353301
**CACNA2D1-AS1**	1.905081571	1.228692474	2.953819501	0.003972394
**AC092168.2**	1.384361407	1.059461203	1.808897297	0.01716253
**AL078645.1**	0.573863049	0.340010232	0.968555556	0.037562158
**AC060809.1**	3.1490413	1.40228695	7.071634735	0.005451042
**AF131215.6**	0.767943581	0.592309903	0.995656735	0.046281614
**LUADT1**	2.728613187	1.076255477	6.917809092	0.034448028
**AC022858.1**	2.7076119	1.183600366	6.193950602	0.018314194
**AC002546.1**	2.070010167	1.128357464	3.797504095	0.018771667
**AL603839.3**	0.728025283	0.534518723	0.991585121	0.044054683
**AC020915.2**	1.569803135	1.051434669	2.343732766	0.027437638
**AC009135.1**	1.911200601	1.216092698	3.003626076	0.004982769
**AC087501.4**	0.627799753	0.408717769	0.964314643	0.033511328
**LINC01537**	2.227880162	1.447058632	3.430026887	0.000274345
**LINC01415**	2.23344205	1.38117306	3.611613586	0.001049506
**AC012050.1**	1.836074169	1.124883452	2.996904567	0.015068819
**PAN3-AS1**	0.693013469	0.483274249	0.993778727	0.046163346

**AL109811.1**	0.458806196	0.223177237	0.943210551	0.03409286
**AC025741.1**	3.482595043	1.604386997	7.559565279	0.001602421
**AC010999.2**	0.420407123	0.23534824	0.75098139	0.00341774
**TMPO-AS1**	1.359042014	1.048004607	1.762392248	0.020691457
**AL354696.2**	2.070114268	1.095901565	3.910363137	0.024950604
**LINC00519**	1.285358951	1.015985778	1.626152322	0.036426459
**ERCC8-AS1**	0.523110471	0.276777049	0.988682286	0.046041928
**AC009318.2**	1.532040725	1.080328092	2.172625892	0.016690238
**AC092111.2**	2.537804515	1.07859431	5.9711531	3.29E-02
**AC104971.3**	0.745066586	0.55821644	0.994460532	0.045752222
**LINC00626**	1.434825675	1.042162422	1.975435569	0.026890084
**AC009318.3**	1.392419564	1.02476461	1.891978143	0.034314593
**AC026355.2**	0.731104658	0.600326267	0.890372537	0.001841238
**AC106038.1**	6.790080033	1.523621638	30.26025997	0.011996396
**L3MBTL2-AS1**	0.651257981	0.424633134	0.998831517	0.049376979
**AC090948.1**	0.701752659	0.516992459	0.952541545	0.023095793
**HLA-DQB1-AS1**	0.84436719	0.737966419	0.966108936	0.013828434
**AL121820.1**	1.550837775	1.027660535	2.340362137	0.036625171
**SMILR**	1.55570094	1.211384808	1.997883248	0.000535393
**AL117335.1**	2.038473264	1.306130704	3.181437535	1.71E-03
**AC105036.3**	1.568948836	1.006699135	2.445219596	0.04665094
**AC079949.1**	1.240771414	1.045542652	1.472454231	0.013517427

**AC127164.1**	1.770475137	1.193012325	2.627451658	0.004565793
**FAM66C**	1.896768368	1.093395668	3.29041933	0.022747445
**AC025419.1**	1.330835024	1.002212311	1.767212237	4.82E-02
**DACT3-AS1**	4.318243572	1.449506363	12.86453652	0.008627357
**LINC00598**	2.429689215	1.406785311	4.196368581	0.001451928
**AP001363.1**	1.469513021	1.089128677	1.982748745	0.011782614
**LINC02360**	1.500859248	1.126165267	2.000220169	0.005592374
**AC012676.4**	0.531198862	0.308406077	0.914937327	0.022582554
**NPAS2-AS1**	2.765663128	1.341092662	5.703478031	0.005874773
**AC108136.1**	1.938636412	1.492379925	2.518334022	7.07E-07
**AC006064.2**	1.497510406	1.033879246	2.169051584	0.03266112
**AL360270.1**	1.556513227	1.131902116	2.140408955	0.006483085
**DLEU1**	1.482858795	1.035872414	2.122723007	0.031356042
**LINC01600**	2.17915349	1.150478452	4.12759572	0.016844464
**AL122035.1**	1.491024239	1.140722502	1.948899297	0.003460534
**AP001528.1**	1.396040882	1.031756838	1.888943278	0.030571484
**AL031666.1**	0.757208566	0.582302268	0.984651519	0.037950691
**AL031600.2**	0.366260709	0.182572894	0.73475807	0.004688917
**FARSA-AS1**	1.663865996	1.054933353	2.624289056	0.02852511
**LINC01776**	2.064036501	1.181386907	3.606140081	0.010912669
**AC087752.3**	0.723638632	0.538731688	0.972010522	0.031671754
**AC090971.2**	1.443250658	1.029438089	2.02340722	3.33E-02

**AC005746.2**	1.732662563	1.178072487	2.54833178	0.005229296
**AL139383.1**	0.443668625	0.202175125	0.973620513	0.042700098
**AL138789.1**	1.538450932	1.216681697	1.945316738	0.000320468
**AC079061.1**	2.925950214	1.648568022	5.193103678	0.000244657
**AC046143.1**	1.607609804	1.116805908	2.314107818	0.010638442
**AC026356.1**	1.403676218	1.066022249	1.848279364	0.015719391
**LINC01747**	3.064244478	1.321746896	7.103927574	0.009049199
**AC012409.4**	1.738352039	1.122705737	2.691593812	0.013181136
**AF121898.1**	1.756979214	1.279410651	2.412810895	0.000496807
**AC007619.1**	1.407740954	1.026153667	1.931225954	3.40E-02
**LINC02310**	2.249583587	1.412947911	3.581608547	0.000633665
**LINC02584**	1.72119216	1.125525587	2.632105824	0.012224565
**AP000695.1**	1.32141095	1.056425221	1.652863699	0.014660503
**ZFHX4-AS1**	1.646934061	1.120857055	2.419926598	0.011051615

Supplementary Table S5. Selecting the CTLA4LncSigs by multivariate Cox regression analysis in the risk score model.

id	coef	HR	HR.95L	HR.95H	p-value
**AP000253.1**	1.608746266	4.996542964	1.406925248	17.74468234	0.012848
**`CACNA2D1-AS1`**	1.125029507	3.080307737	1.631548439	5.815515819	0.000521
**AL078645.1**	-0.906333235	0.404002895	0.213385517	0.764898863	0.005388
**LINC01415**	0.662403301	1.939447815	1.131752718	3.323568629	0.015938
**AC010999.2**	-1.187922481	0.304853947	0.140602297	0.660984429	0.002625
**`ERCC8-AS1`**	-1.349894184	0.259267694	0.110058933	0.610761301	0.002017
**AC104971.3**	-0.454416546	0.634818247	0.467873773	0.861331047	0.003514
**AC026355.2**	-0.203542909	0.815835197	0.653289611	1.018823898	0.07258
**`L3MBTL2-AS1`**	-0.59542681	0.551327204	0.329648128	0.922079212	0.023261
**AL117335.1**	0.674354484	1.96276557	1.139696975	3.380239458	0.015039
**AC105036.3**	0.691925884	1.997558897	1.066091127	3.742870985	0.030794
**AC127164.1**	0.964229071	2.622764912	1.297796679	5.300441811	0.007229
**FAM66C**	0.761046206	2.140514469	1.051819052	4.356074538	0.035788
**`NPAS2-AS1`**	1.266421106	3.548131427	1.19053607	10.57442688	0.023027
**AC108136.1**	0.41175641	1.5094667	1.121291419	2.032022792	0.006633
**AL031600.2**	-0.887648317	0.411622621	0.178718774	0.948043561	0.037041
**`FARSA-AS1`**	0.638161068	1.892996585	1.1238825	3.188443694	0.01644
**AL139383.1**	-0.735879109	0.479084107	0.186648203	1.229701534	0.126006
**AC079061.1**	1.126432975	3.084633886	1.546130204	6.154052346	0.001391

Supplementary Table S6. The result of pleiotropic test.

id.exposure	id.outcome	egger_intercept	se	pval
ebi-a-GCST90103632	ebi-a-GCST004748	-0.00159	0.001975	0.420487

"id.exposure" means the exposure ID in IEU Open GWAS platform; "id.outcome" means the outcome ID in IEU Open GWAS platform; "se" means standard error of effect size; "pval" means p-value of statistical significance.

Supplementary Table S7. Five methods of 2SMR.

id.exposure	id.outcome	method	nsnp	b	se	pval	lo_ci	up_ci	or	or_lci95	or_uci95
ebi-a-GCST90103632	ebi-a-GCST004748	Inverse variance weighted	328	-0.416670621	0.208488796	0.045659487	-0.825308662	-0.00803258	0.659238024	0.438099747	0.991999595
ebi-a-GCST90103632	ebi-a-GCST004748	MR Egger	328	-0.103247414	0.441026569	0.815048851	-0.967659489	0.76116466	0.901903802	0.379971325	2.140768037
ebi-a-GCST90103632	ebi-a-GCST004748	Weighted median	328	-0.397367248	0.310367119	0.200434861	-1.005686801	0.210952304	0.672087158	0.365793321	1.234853457
ebi-a-GCST90103632	ebi-a-GCST004748	Simple mode	328	-0.311746208	0.737237566	0.672677394	-1.756731838	1.133239422	0.732167323	0.172608054	3.105700897
ebi-a-GCST90103632	ebi-a-GCST004748	Weighted mode	328	-0.229895173	0.373429398	0.538565515	-0.961816793	0.502026447	0.794616895	0.38219788	1.652065704

Open GWAS platform; "nsnp" means the number of using SNP; "b" means the estimate of the effect size; "se" means standard error of effect size; "pval" means p-value of statistical significance; "lo_ci" means lower bound confidence interval for the estimate; "up_ci" means upper confidence interval for the estimate; "or" means odds ratio; "or_lci95" means 95% lower bound confidence interval for the odds ratio; "or_uci95" means 95% upper confidence interval for the odds ratio.
